# Increased antibiotic resistance gene abundance linked to intensive bacterial competition in the phyllosphere across an elevational gradient

**DOI:** 10.1111/1758-2229.70042

**Published:** 2024-11-21

**Authors:** Yihui Ding, Rui‐Ao Ma, Ran Zhang, Hongwei Zhang, Jian Zhang, Shaopeng Li, Si‐Yu Zhang

**Affiliations:** ^1^ School of Ecological and Environmental Sciences East China Normal University Shanghai China; ^2^ School of Life Sciences Sun Yat‐Sen University Guangzhou China

## Abstract

Antibiotic resistance genes (ARGs) are ancient and widespread in natural habitats, providing survival advantages for microbiomes under challenging conditions. In mountain ecosystems, phyllosphere bacterial communities face multiple stress conditions, and the elevational gradients of mountains represent crucial environmental gradients for studying biodiversity distribution patterns. However, the distribution patterns of ARGs in the phyllosphere along elevational gradients, and their correlation with bacterial community structures, remain poorly understood. Here, we applied metagenomic analyses to investigate the abundance and diversity of ARGs in 88 phyllosphere samples collected from Mount Tianmu, a national natural reserve. Our results showed that the abundance of ARGs in the phyllosphere increased along elevational gradients and was dominated by multidrug resistance and efflux pumps. The composition of bacterial communities, rather than plant traits or abiotic factors, significantly affected ARG abundance. Moreover, increased ARG abundance was correlated with greater phylogenetic overdispersion and a greater proportion of negative associations in the bacterial co‐occurrence networks, suggesting that bacterial competition primarily shapes phyllosphere resistomes. These findings constitute a major advance in the biodiversity of phyllosphere resistomes along elevations, emphasizing the significant impact of bacterial community structure and assembly on ARG distribution, and are essential for understanding the emergence of ARGs.

## Graphical abstract

Minimal research has been conducted on the phyllosphere resistome in relatively pristine environments. This study reports an increase in the abundance of phyllosphere resistomes along an elevational gradient in a national natural reserve, with implications for the spread of antibiotic resistance genes (ARGs).

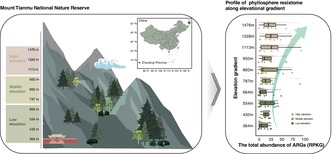

## INTRODUCTION

Antibiotic resistance genes (ARGs) are not only a growing global concern (Hernando‐Amado et al., 2019) but also an ancient phenomenon that has existed and evolved over millions of years (D'Costa et al., 2011). It is intrinsic to various natural habitats, including aquatic (Chen, Li, et al., 2019), glacial (Sajjad et al., 2023), terrestrial (Tang et al., 2015), air (Zhu et al., 2021) and phyllosphere (Li et al., 2023) environments. ARGs in natural habitats have been found to provide survival advantages against antimicrobial agents and toxic compounds produced by microbes (Darby et al., 2023), thereby enhancing bacterial fitness and adaptation to challenging conditions (Cruz‐Loya et al., 2019). Under various stresses, such as nutrient limitation and low temperature, bacteria have been shown to secrete antibiotics/antimicrobials to inhibit or kill other competitors and thus select antibiotic‐resistant bacteria (Bahram et al., 2018).

The phyllosphere is the aerial part of plants, and the surface area of the phyllosphere is estimated to be more than 10^9^ km^2^ globally (Vorholt, 2012). Phyllosphere bacterial communities face a challenging environment characterized by limited nutrient availability, UV radiation and low water availability, leading to diverse biochemical and metabolic adaptations that enable survival under these multiple stress conditions (Schlechter et al., 2023; Trivedi et al., 2020). Biotic factors, such as microbial interactions for competing nutrients through decreasing niche differences and increasing fitness between competing species, play an important role in shaping variances in phyllosphere bacterial communities (Schlechter et al., 2023). Moreover, the phyllosphere has been reported to be one of the most important reservoirs of ARGs in terrestrial ecosystems (Zhu et al., 2022). The abundances of ARGs in the phyllosphere are mostly attributed to air pollution, population aggregation and industrialization (Huang et al., 2023; Yan et al., 2020). However, these studies were mostly conducted in urban greenspaces, which are predominantly affected by anthropogenic activities. Along with geographic patterns, the phyllosphere resistome is sensitive to changes in temperature during climate warming (Li et al., 2022; Zhou et al., 2023). Additionally, biotic factors, such as bacterial community composition, are also the main factors shaping phyllosphere resistomes (Yan et al., 2021). Nevertheless, whether the variance in phyllosphere resistomes is associated with the bacterial community assembly is still poorly understood due to the complex environmental factors involved.

Mountain ecosystems play an important role in maintaining Earth's biodiversity and provide multiple vital functions in the nutrient cycle and climate regulation (Rahbek, Borregaard, Antonelli, et al., 2019; Rahbek, Borregaard, Colwell, et al., 2019). The elevational gradients in mountain ecosystems represent one of the intricate ways in which species adapt to climate change by shifting their distribution and ranges (Chen et al., 2009). This response creates a unique “experiment by nature” for studying the mechanisms driving the evolution and maintenance of biodiversity (Lomolino, 2001; Payne et al., 2017). Elevational gradients offer a unique opportunity to explore comprehensive environmental changes over shorter distances, enabling detailed studies on how shifts in abiotic and biotic factors impact the biodiversity patterns of phyllosphere bacterial communities and their functional genes (Nogues‐Bravo et al., 2008). Although the biodiversity of plants and animals along elevational gradients has been widely studied (Albrecht et al., 2021; Grytnes & McCain, 2007; Wang et al., 2022), less is known about microbial diversity, particularly in the phyllosphere, than about soil bacterial communities (Dai et al., 2021; Hu et al., 2020; Wang et al., 2022).

Accumulating studies have indicated that a decrease in temperature and resource availability along elevational gradients (Barry, 2008; Mayor et al., 2017) could affect the assembly pattern of the microbial community (Nottingham et al., 2018; Wang et al., 2022) and thus result in increased levels of negative interactions (such as competition) in the phyllosphere bacterial community (Schafer et al., 2022; Schlechter et al., 2023). We hypothesized that competition among phyllosphere bacterial communities will be more intense at higher elevations owing to harsh environmental conditions. Moreover, intensified bacterial interactions may lead to or be associated with a greater abundance of ARGs. However, to date, bacterial assembly along elevational gradients and the distribution patterns of phyllosphere ARGs have rarely been explored. Herein, we chose a subtropical natural reserve, which is a relatively pristine environment with little anthropogenic activity, to explore the distribution pattern of phyllosphere ARGs and their associations with phyllosphere bacterial communities. We aimed to answer the following questions: (i) What are the biodiversity and bacterial community structures in the phyllosphere along elevational gradients? (ii) What are the differences in the abundance and diversity of phyllosphere ARGs along the elevational gradients? (iii) Does bacterial community structure and assembly contribute to the variances in phyllosphere resistomes?

## EXPERIMENTAL PROCEDURES

### Study area and leaf sampling

Mount Tianmu National Nature Reserve is a subtropical forest located on the northwest of Hangzhou, Zhejiang province (30°18′30″–30°21′37″ N, 119°24′11″–119°27′11″ E), with minimal human impacts. Between 28 August and 1 September, 2022, a total of 88 samples were collected from 23 tree species (affiliated with 11 plant families) across 10 elevations in the Mount Tianmu National Nature Reserve. At each elevation, leaves were sampled from three dominant plant species in triplicate (three individual trees), for a total of nine leaf samples for each elevation. However, only seven leaf samples were collected at 432 m because only one leaf sample was obtained from the plant species *Maclura tricuspidate* (Table S1). Fifteen grams of healthy leaves that were not subjected to direct ultraviolet (UV) radiation were collected from each tree. All the leaves were collected using ethanol‐sterilized scissors, stored in sterilized bags and kept in a low‐temperature storage area at 4°C. The plant traits of 23 plant species, including leaf area, LDMC (leaf dry matter content), leaf chemical content (leaf C, N and P concentrations) and soil properties (soil nitrogen content, soil carbon, soil phosphate content, soil moisture content and soil pH), were collected at every elevation and are detailed in the Supporting Information. The map of the specific location of 10 sampling sites was generated via Google Earth Pro (https://earth.google.com/). The 88 samples were grouped into three groups distributed at low (364–664 m; 34 samples), middle (787–950 m; 27 samples) and high elevations (1113–1476 m; 27 samples) considering the combined variances of soil properties, air temperature and leaf traits (including leaf dry matter content, leaf area, leaf carbon content, leaf nitrogen content and leaf phosphorus content), as well as vegetation types across different elevations (detailed in the Supporting Information).

### Shotgun metagenomic sequencing, quality control and metagenome‐assembled genome (MAG) binning

Microbial DNA was extracted from ~15 g of leaves, and good‐quality DNA was obtained for sequencing on the Illumina sequencing platform at Majorbio Bio‐Pharm Technology Co., Ltd. (Shanghai, China). Details are provided in the Supporting Information. All the raw metagenome sequencing reads were trimmed for quality by multitrim (version 1.2.6) with default settings (https://github.com/KGerhardt/multitrim). Chloroplast sequences from trimmed sequences were removed via BLAT version 2.3.4.1 (Kent, 2002) against plant chloroplast genomes from the National Center for Biotechnology Information (NCBI) database. The removal of plant and homologous sequences was performed with Kraken2 (Lu et al., 2022) (version 2.0.7_beta) and Krakentools (extract_kraken_reads.py ‐include‐children). Trimmed and clean metagenomic sequencing paired reads were assembled by MEGAHIT version 1.2.9 with default settings (Li et al., 2016) and contigs longer than 1500 bp were subsequently used for the binning of metagenome‐assembled genomes (MAGs) via MetaBAT2 version 2.15 (Kang et al., 2019) and MaxBin2 version 2.2.7 (Wu et al., 2016). The calculations of the relative abundance, taxonomic classification and functional annotation of the MAGs are detailed in the Supporting Information.

### 16S rRNA gene extraction and taxonomic classification

16S rRNA sequences were extracted from metagenomic reads by SortMeRNA (v.4.3.4) (Kopylova et al., 2012) using the silva‐bc‐16 s‐id90.fasta and silva‐arc‐16 s‐id95.fasta databases (Quast et al., 2012) with the settings ‐fastx ‐paired_out ‐e value 1e‐5. All the extracted 16S rRNA sequences were analysed by QIIME 2‐vsearch (Bolyen et al., 2019) using a closed reference search against SILVA 138 to generate operational taxonomic unit (OTU) tables based on 97% similarity. All the bacterial OTUs were annotated by the Qiime2 feature classifier against silva‐138‐99‐seqs.qza and silva‐138‐99.tax.qza based on an identity of 99%. Eukaryotic, mitochondrial and chloroplast‐derived OTUs were filtered and removed before further analysis. The differentially abundant bacterial OTUs between paired elevation gradients were assessed by the package “DESeq2” (Love et al., 2014) based on the unrarefied OTUs table (FDR‐adjusted *p* < 0.01, |log2fold change| > 2).

### Functional annotation of ARGs

Antibiotic resistance genes were annotated by BLASTx of DIAMOND (version 2.014.152) (Buchfink et al., 2015) of the clean reads against the SARG database (Yin et al., 2018). The best hit for each query was extracted using BlastTab.best_hit_sorted.pl (http://enve-omics.ce.gatech.edu/enveomics/docs?t=BlastTab.best_hit_sorted.pl/) and filtered with a cut‐off of identity of ≥ 90%, alignment length of ≥ 25 amino acids and e‐value of ≥ 1e‐7. For the annotation of ARGs of MAGs, BLASTp (Altschul et al., 1990) was used with a cut‐off of identity of ≥ 50% and a query length coverage of ≥ 50%. The relative abundance of ARGs was normalized as reads per kilobase per genome equivalent (RPKG), which indicates the fraction of total cells encoding the gene of interest, that is, copies per cell (Zhang et al., 2021). The RPKG was calculated as follows: RPKG = (reads mapped to gene)/(gene length in kb)/(genome equivalents).

### Null model analysis and co‐occurrence network construction

The phylogenetic structures of phyllosphere bacterial communities were assessed via standardized effect sizes (SES) of mean pairwise distance (MPD). First, the phylogenetic tree of all phyllosphere bacterial OTUs was constructed with QIIME 2 via the q2‐phylogeny plugin (align‐to‐tree‐MAFFT‐fasttree pipeline). Second, the MPD among OTUs in a local community was calculated on the basis of the phylogenetic tree via the “mpd” function in the “vegan” package. Finally, SESmpd, which examines whether co‐occurring taxa have closer (negative values) or more distant (positive values) phylogenetic relationships than expected by chance, was calculated based on a null model (null.model = “taxa.labels,” iterations = 9999) with the function “ses.mpd” in the package “picante” as follows: MPDobs‐MPDrand/sd_MPDrand (Kembel et al., 2010).

A co‐occurrence network of bacteria was constructed using the “igraph” (Csardi, 2013) and “psych” (Revelle & Revelle, 2015) packages in R v4.2.3 and visualized via the Gephi platform (https://gephi.org/). The rarefied OTU table (read counts) was used, and bacterial OTUs with occurrence in at least 20% of the samples in each elevational group were retained for network analysis. The pairwise Spearman's correlations between OTUs were calculated, with an absolute correlation coefficient ≥ 0.6 and a corrected *p* value < 0.01 (Benjamini–Hochberg procedure) considered valid relationships (Benjamini & Hochberg, 1995). The topological features, including nodes, edges, average degree, modularity, positive correlations, negative correlations, degree (the number of adjacent edges) and betweenness centrality (the number of shortest paths passing through a node), were calculated. In addition, the subnetwork of each sample was extracted from the metacommunity co‐occurrence network to examine the differences in the proportions of positive and negative correlations between OTUs at low, middle and high elevations. The clustering coefficient of subnetworks was calculated to analyse the contribution of bacterial interactions and environmental variables to ARG abundance. An interkingdom network was constructed to evaluate the co‐occurrence pattern of ARGs and OTUs and visualized via the Cytoscape platform (https://cytoscape.org/). Bacterial OTUs with a frequency of < 0.2 and ARG subtypes with a frequency of < 0.3 were removed first before construction of the network, and ARGs and OTUs with correlation coefficients of ≥0.6 and corrected *p* < 0.01 (Benjamini–Hochberg procedure) were included in the interkingdom network.

### Statistical analysis

The rarefied OTU table of bacterial communities and counts of ARGs were used to calculate the alpha diversity indices. Alpha diversity (measured by richness and Shannon indices) and beta diversity (nonmetric multidimensional scaling (NMDS) ordination based on Bray–Curtis dissimilarity) of both bacterial communities and functional genes across phyllosphere samples were calculated via the “vegan” package (Oksanen et al., 2007). The first axis value of NMDS (NMDS1) of the bacterial community was used to represent the variances in bacterial community compositions. A phylogenetic tree of the 23 plant species (affiliated with 11 plant families) was constructed by mega‐tress in the package “rtrees” (Li, 2023). The phylogenetic distances between pairs of plant species were calculated using the pairwise distances from the species' phylogenetic tree, via the function “cophenetic.phylo” in the package “ape” (Paradis et al., 2004), and were decomposed into eigenvectors via the function “pcnm” in the package “vegan” (Table S7). For the variances in plant traits (leaf surface area, leaf dry matter content, leaf nitrogen content, leaf carbon content and leaf phosphate content) and soil properties (the total content of carbon, nitrogen and phosphate, soil pH and moisture contents), principal component analysis (PCA) was performed, and the first axis values (PC1) were used to represent the variances in plant traits (Table S5) and soil properties (Table S6). Canonical correspondence analysis (CCA) and variance partitioning analysis (VPA) were used to evaluate the contributions of multiple factors to the variation in the bacterial community structure. Spearman correlation was performed to explore the relationships between biotic (bacterial NMDS1) and abiotic factors and ARG abundance. Other statistical analyses, including PERMANOVA, Procrustes analysis and Mantel tests, were also carried out and are detailed in the Supporting Information. All statistical analyses and graphics were performed in the R environment (version 4.2.3).

### Structural equation model (SEM) construction

The structural equation model (SEM) was generated via the “lavaan” (Rosseel, 2012) package to evaluate the direct and indirect impacts of biotic factors (i.e., bacterial composition) and abiotic factors (i.e., air temperature and plant and soil properties) on ARG abundance. Variables, including the air temperature at each elevation, plant phylogeny (PCNM1; Tables S7 and S8) and variance in soil properties (PC1; Table S6), plant traits (PC1; Table S5) and bacterial communities (NMDS1; Table S4), were included in the SEM to investigate different hypothetical pathways that contribute to ARG abundance. In the SEM, the air temperature was the key exogenous variable. Plant attributes (including plant phylogeny and plant traits), soil properties and the bacterial community were the endogenous explanatory variables. In addition, ARG abundance was the endogenous variable. The general linear model with a quadratic term was used to assess potential nonlinear relationships between exogenous and endogenous variables and revealed no nonlinearities between different variables. The *p* value, GFI (goodness‐of‐fit index) and RESEA (root‐mean‐square error of approximation) were used to evaluate the model.

## RESULTS

### Overview of phyllosphere samples and bacterial communities

**FIGURE 1:**
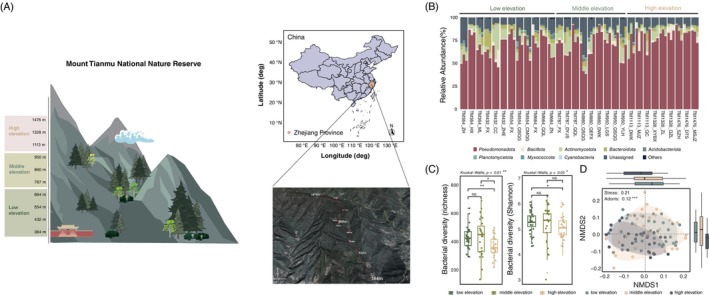
Study area and structure of the phyllosphere bacterial communities. (A) All samples were collected from Mount Tianmu National Nature Reserve. (B) The composition of the phyllosphere bacterial communities at the phylum level along the elevational gradient. (C) Alpha diversity was determined on the basis of the richness and Shannon index in the three elevation groups. * and ** above the paired elevation gradients indicate that the Wilcoxon rank‐sum test *p* < 0.05 and *p* < 0.01, respectively, for the multigroup Kruskal–Wallis test. (D) Non‐metric multidimensional scaling (NMDS) based on Bray–Curtis dissimilarity. The symbol *** indicates PERMEANOVA: *p* < 0.001.

In total, 34 samples were collected at low elevations (364–664 m) and 27 samples were collected at both middle (787–950 m) and high elevations (1113–1476 m; Figure 1A). Approximately 5 Gbp of shotgun metagenomic data were acquired for each of the phyllosphere samples. The coverage of the 88 metagenomes achieved by sequencing ranged from 50.03% to 93.38% (Table S2), as assessed by Nonpareil 3.0 (Rodriguez‐R & Konstantinidis, 2013), which is adequate for between‐sample comparisons and assembly. At the phylum level, the phyllosphere bacterial communities in Mount Tianmu were dominated by *Pseudomonadota* (72.1%), which was mostly composed of *Acinetobacter*, *Pseudomonas*, *Pantoea*, *1174‐901‐12* and *Methylobacterium–Methylorubrum*, followed by *Actinomycetota* (7.5%), *Bacteroidota* (3.6%) and *Bacillota* (2.4%; Figure 1B and Figure S4). The diversity of the bacterial communities (richness and Shannon index) varied significantly (Kruskal–Wallis, *p* = 0.01) along the elevational gradient (low, middle and high elevations), and the greatest richness occurred at the middle elevations (Figure 1C). A comparison of phyllosphere bacterial composition performed using nonmetric multidimensional scaling (NMDS) ordination indicated significant variations in bacterial communities at different elevations (PERMANOVA, *p* < 0.001; Figure 1D). Additionally, a total of 71 bacterial OTUs belonging to 2 phyla (*Pseudomonadota* and *Actinomycetota*) and 22 genera were identified with significantly different abundances among the three elevations (Table S3). Among them, 27 bacterial OTUs belonging mainly to *Pseudomonadota* (i.e., *Pseudomonas*, *Acinetobacter* and *Ralstonia*) were enriched at low elevations. The majority of the differential bacterial OTUs belonging to *Actinomycetota* were enriched at middle elevations. The 21 bacterial OTUs enriched at high elevations were mainly affiliated with *Pseudomonadota*, such as *Methylobacterium–Methylorubrum*, *Sphingomonas* and *Serratia* (Figure S5).

### Bacterial assembly along the elevational gradient

**FIGURE 2:**
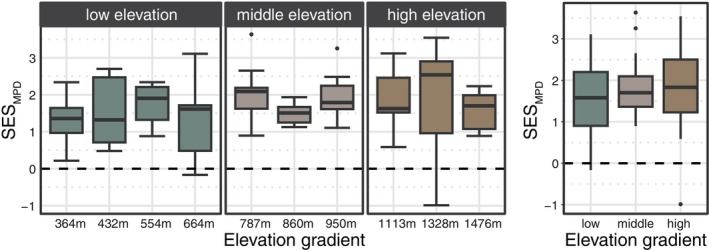
The community assembly of natural phyllosphere bacterial communities along an elevational gradient. The standardized effect sizes (SES) of the mean paired distance (MPD) value for evaluating the phylogeny of bacterial communities based on the “taxa. labels” null model.

SESmpd in each local community was used to explore the community assembly of phyllosphere bacterial communities, and a highly positive SESmpd value indicates that the bacterial communities are more distantly related, suggesting phylogenetic overdispersion (Webb et al., 2002). Positive SESmpd values were detected at all elevations, and the highest SESmpd value was detected at high elevations (Figure 2). Overall, the SESmpd value at high (1.84 ± 0.98) and middle (1.82 ± 0.64) elevations was greater than that at low (1.54 ± 0.84) elevations, although no significant (*p* > 0.05) difference was revealed between each group. A series of analyses showed that environmental factors and plant traits could seldom explain the variations in phyllosphere bacterial communities along elevational gradients (9.9%), and the majority of the variations (90.1%) remained unexplained (Figure S6).

**FIGURE 3:**
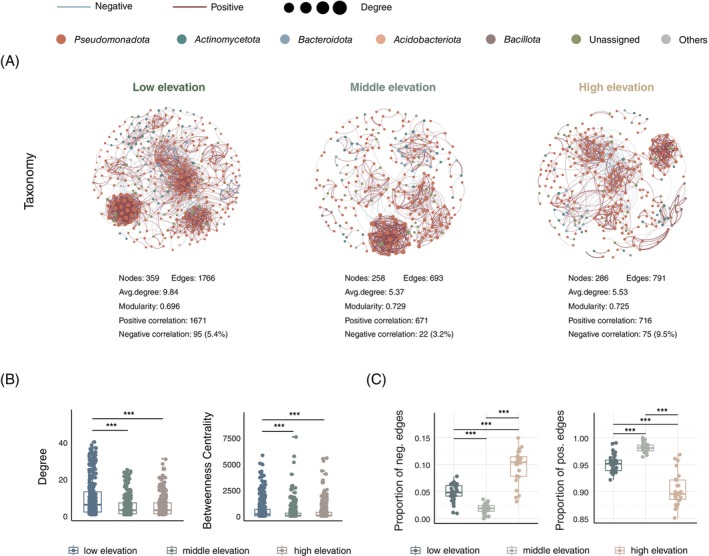
Ecological co‐occurrence network for bacterial interaction patterns. (A) The bacterial interaction network is distinguished by taxonomy. (B) Degree and betweenness centrality of the network. (C) The proportion of negative and positive edges in the individual sample subnetwork.

The co‐occurrence network of bacteria at low, middle and high elevations was further constructed to investigate the interactions among phyllosphere bacterial communities. Accordingly, greater numbers of nodes (359) and edges (1766) were detected at low elevations than at moderate (nodes of 258 and edges of 693) and high (nodes of 286 and edges of 791) elevations (Figure 3A). The degree and betweenness centrality of the co‐occurrence network at low elevations were significantly greater than those at moderate (Wilcoxon rank‐sum test, *p* < 0.001) and high elevations (Wilcoxon rank‐sum test, *p* < 0.001; Figure 3B). However, the proportion of negative edges to total edges at high elevations (9.5%) was greater than that at low (5.4%) and middle elevations (3.2%; Figure 3A). Moreover, the proportions of negative associations to total associations in the subnetwork at high elevations were significantly greater than those at low (Wilcoxon rank‐sum test, *p* < 8.97e‐09) and middle elevations (Wilcoxon rank‐sum test, *p* < 4.11e‐15; Figure 3C). These bacterial taxa in the bacteria–bacteria co‐occurrence networks were mainly affiliated with *Pseudomonadota* (65.4%–74.5%), *Actinomycetota* (8.5%–9.7%), *Bacteriadota* (3.8%–7.2%), *Acidobacteriota* (0.3%–3.6%) and *Bacillita* (0.8%–2.1%) at the phylum level (Figure 3A).

### Characteristics of phyllosphere resistomes along the elevational gradient

**FIGURE 4:**
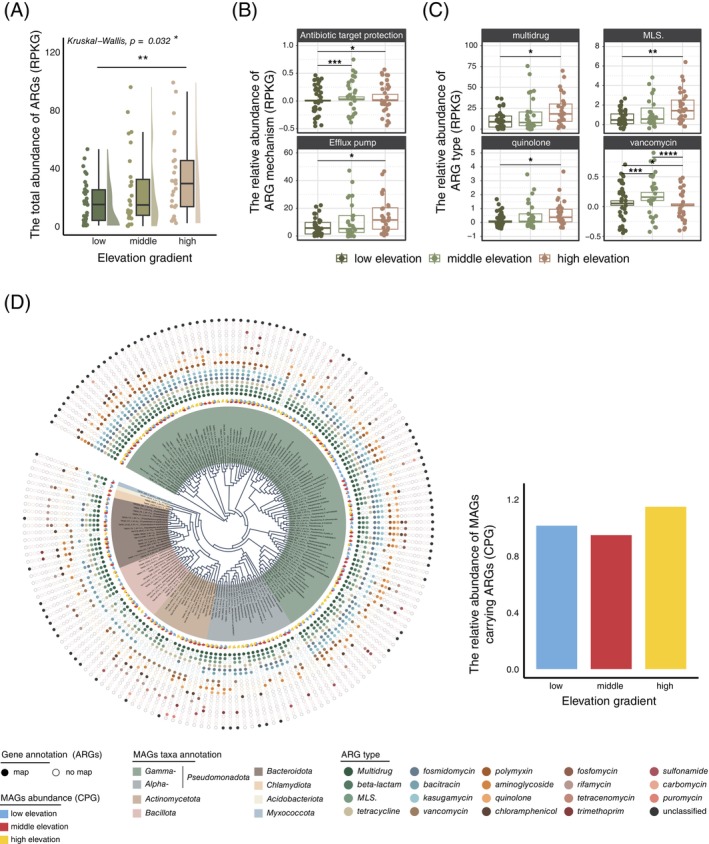
Characterization of phyllosphere ARGs along the elevational gradient. (A) The relative abundance of ARGs at different elevations. Kruskal–Wallis analysis was used for the global test and the Wilcoxon rank‐sum test was used for the paired group test. * and ** indicate *p* values <0.05 and <0.001, respectively. The relative abundance of ARG (B) mechanisms and (C) types. For the Wilcoxon rank‐sum test, *, **, *** and **** indicate *p* < 0.05, *p* < 0.01, *p* < 0.001 and *p* < 0.0001, respectively. (D) Phylogenic tree of the recovered genomes from the phyllosphere metagenomes.

Overall, the highest abundance of phyllosphere ARGs was detected at high elevations (Figure S7), which was significantly greater than that of ARGs at low elevations (Wilcoxon rank‐sum test, *p* < 0.01; Figure 4A). Multidrug resistance was the predominant ARG type identified in the phyllosphere resistomes, accounting for 66.74% of the total ARG abundance (Figure S8A). While the abundance of ARGs increased with increasing elevation, the richness of ARGs decreased at high elevations (Figure S8B). Moreover, a clear separation pattern of ARGs among different elevations was revealed by NMDS (PERMANOVA, *p* < 0.05; Figure S8C). Specifically, the relative abundance of ARGs encoding efflux pumps and antibiotic target protection agents was significantly greater at high elevations than at low elevations (Wilcoxon rank‐sum test, *p* < 0.05; Figure 4B). Among the different ARG types, multidrug, macrolide‐lincosamide‐streptogramin (MLS) and quinolone resistance increased in abundance along the elevational gradient (Wilcoxon rank‐sum test, *p* < 0.05; Figure 4C). However, the relative abundance of vancomycin was significantly greater at middle elevations than at low and high elevations (Wilcoxon rank‐sum test, *p* < 0.001; Figure 4C). ARG richness and abundance were significantly, either positively or negatively, correlated with the significantly differentially abundant bacteria *Pseudomonadota* and *Actinomycetota* (Figure S9A; detailed in the Supporting Information).

In total, 172 MAGs with completeness > 50% and contamination < 10% were obtained. Approximately 97.7% of the MAGs (169/172) carried at least one ARG type in the genome, and the relative abundance of MAGs carrying ARGs at high elevations was greater than that at low and middle elevations (Figure 4D and Table S9). Among them, multidrug, beta‐lactam, MLS, tetracycline, fosmidomycin, bacitracin and kasugamycin resistance were the dominant ARG types, and over 95.4%, 76.2%, 78.0%, 69.2%, 62.2%, 68.6% and 53.5% of MAGs carried these ARG types, respectively. MAGs carrying these dominant resistance genes were more abundant at high elevations than at low and middle elevations (Figure S10). According to the taxonomic classification, these MAGs were mainly affiliated with *Pseudomonadota* (*n* = 123), including *Gammaproteobacteria* and *Alphaproteobacteria* at the class level, followed by *Bacteroidota* (*n* = 17), *Actinomycetota* (*n* = 15) and *Bacillota* (*n* = 14).

### Co‐occurrence of phyllosphere bacterial communities and ARGs

**FIGURE 5:**
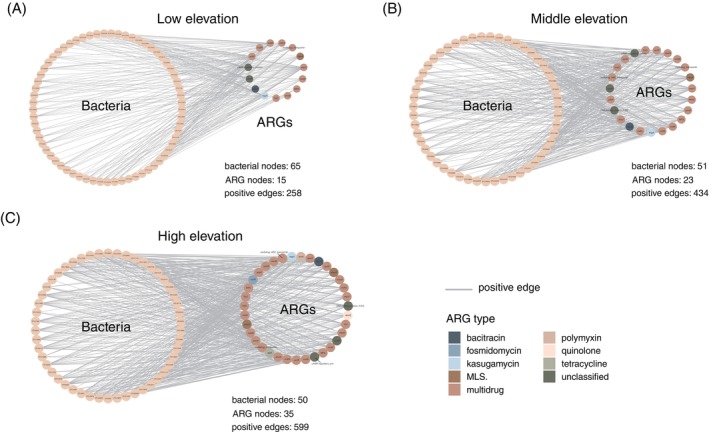
Co‐occurrence network showing the associations of ARGs and bacterial OTUs. (A), (B) and (C) represent the co‐occurrence networks for low elevations, middle elevations and high elevations, respectively.

An interkingdom network for ARG subtypes and bacterial OTUs at each elevation (low, middle and high) was constructed to explore the relationships between phyllosphere bacterial communities and ARGs. An increased number of ARG subtypes was detected along the elevational gradient in the network, that is, 15, 23 and 35 for low, middle and high elevations, respectively (Figure 5). These ARG subtypes were mainly multidrug resistance genes, accounting for approximately 66.6%–73.9% of the total observed ARGs (Figure S11A). The positive associations between bacteria and ARGs also gradually increased along the elevational gradient, that is, 258, 434 and 599 for low, middle and high elevations, respectively (Figure 5). Among these positive associations, *Pseudomonadota* (86.2%–92.0%), which comprises the *Acinetobacter* (38.5%–60.8%) genus, was the predominant bacterial phylum, followed by *Enterobacter* (6.0%–9.8%) and *Pseudomonas* (0%–7.8%; Figure S11B). The abundance of quinolone resistance (*abaQ*), polymyxin resistance (*arnA*) and tetracycline resistance (*tetR*) genes was greater at high elevations (0.59, 0.36 and 0.62 RPKG) than at low elevations (0.23, 0.20 and 0.24 RPKG; Figure S12). Most bacteria that had a positive association with *arnA* genes were assigned to the *Enterobacteriaceae* and *Erwiniaceae* families (Table S10). Moreover, six bacterial OTUs whose abundance significantly increased at high elevations (Figure S5D) were confirmed to be positively correlated with polymyxin resistance (*arnA*; Figure 5C). The bacteria that showed a positive association with the *abaQ* (Table S11) and *tetR* (Table S12) genes were affiliated with the *Acinetobacter* genus.

### Biotic and abiotic factors contributing to the variations in ARG abundance

**FIGURE 6:**
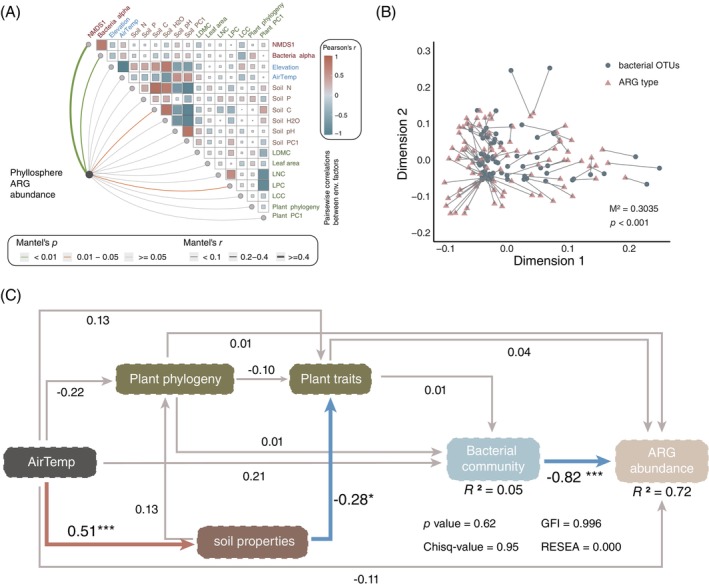
Biotic factors and abiotic factors contribute to the variations in ARG abundance. (A) Mantel test based on Bray–Curtis and Euclidean distances. The colour of the lines indicates the *p* value, and the width of the lines indicates Mantel's *r*. (B) Procrustes test between bacterial OTUs and ARGs based on PCA. (C) Structural equation model (SEM) illustrating the direct and indirect factors that drive the variations in ARG abundance along the elevational gradient. The grey lines represent path coefficients with no significance, the blue lines represent negative path coefficients with significance and the red lines represent positive coefficients with significance.

The abundance of ARGs in the phyllosphere was most affected by the bacterial composition (*r* > 0.4, *p* < 0.01), followed by the soil carbon content (*r* < 0.1, 0.01 < *p* < 0.05) and leaf phosphate content (mantel test, *r* < 0.1, 0.01 < *p* < 0.05; Figure 6A). Similarly, according to the Procrustes analysis results, the phyllosphere ARG profile was significantly correlated with the phyllosphere bacterial community structure (*M* ^2^ = 0.3035, *p* < 0.001; Figure 6B). Bacterial community composition and alpha diversity were significantly (*p* < 0.001) positively correlated with ARG abundance and elevation, and leaf traits, including leaf dry matter content (LDMC) and leaf carbon content (LCC), exhibited weak correlations with ARG abundance (*p* < 0.05; Table S13). A structural equation model (SEM) was constructed to further explore the direct and/or indirect relationships among environmental variables, plant factors (trait and phylogeny) and bacterial composition (NMDS1 value) that influence ARG abundance in the phyllosphere (Figure 6C). A nonsignificant chi‐square *p* value of 0.62 and a low RMSEA (0.000) were obtained for the SEM, which explained 72% of the variance in ARG abundance along the elevational gradient. The variance in the bacterial communities was the significant variable that directly affected the ARG abundance (*p* < 0.001). The direct influence of other variables, including air temperature, plant phylogeny and plant traits, on ARG abundance was also analysed but no significant contribution to ARG abundance variance was revealed. Other factors, including air temperature, plant factors and soil properties, contributed to the variance in ARG abundance indirectly through direct influences on the bacterial community (Figure 6C).

## DISCUSSION

The phyllosphere bacterial richness and diversity in the Tianmu Mountain National Nature Reserve (364–1476 m) showed a hump pattern (Figure 1C), which is also one of the typical distribution patterns observed for plant and animal diversity (Antonelli et al., 2018; McCain & Grytnes, 2010). Intermediate elevations, which harbour less moisture and temperature limitations, and greater soil contents of carbon, nitrogen and phosphorus compared to that at low and high elevations (Figure S1) possibly resulted in the increased bacterial diversity (Dai et al., 2021; Dudney et al., 2023). In addition to the climate factors (temperature and moisture) and soil prosperities, plant attributes could also contribute to the distribution pattern of bacteria in mountain ecosystems (Laforest‐Lapointe et al., 2016; Wang et al., 2023). However, our results showed that environmental factors and plant traits seldom explained the variations in phyllosphere bacterial communities along elevational gradients (9.9%; Figure S6). Consistently, SEM revealed that environmental and plant factors explained only a limited portion of the variation in phyllosphere bacterial communities (*R* ^2^ = 0.05; Figure 6C). Moreover, recent studies have also corroborated the limited impact of environmental and plant attributes on the variation in phyllosphere bacterial community composition (Wang et al., 2023; Yang et al., 2023).

According to the co‐occurrence subnetwork of bacterial communities, a significantly (*p* < 0.001; Figure 3C) greater proportion of bacteria with negative associations was further revealed at high elevations than at low and middle elevations, indicating that more negative interactions between bacteria occurred at high elevations. The negative interaction among most phyllosphere bacteria has been previously reported due to nutrient limitations in the phyllosphere (Schafer et al., 2022). Nevertheless, recent studies also argued that the correlation revealed in the co‐occurrence network could not be interpreted as a signal of biotic interactions (Blanchet et al., 2020). Overall, ~20% of cooccurrences in the network could represent interactions (Cazelles, 2024), and the species distribution and co‐distribution pattern could also be driven by other potential non‐measured environmental factors (Blanchet et al., 2020). Therefore, in addition to negative bacterial interactions, the large percentage of unexplained variation (90.1%; Figure S6) in phyllosphere bacterial communities could also be attributed to the dispersal of bacteria from neighbouring plants (Meyer et al., 2022), plant secondary metabolites (i.e., volatile organic compounds and antimicrobials) (Zaynab et al., 2018; Zelezniak et al., 2015) and other unknown environmental variables that were not assessed.

The positive SESmpd value observed for the bacterial communities indicated that the bacterial community was experiencing greater phylogenetic overdispersion (Webb, 2000; Webb et al., 2002). Moreover, the increased SESmpd value at high and middle elevations (Figure 2) possibly suggested a relatively greater contribution of competition in bacterial community assembly at high and middle elevations than at low elevations. Microbial interactions at high latitudes have been confirmed to be important factors in shaping the distribution of ARGs (Delgado‐Baquerizo et al., 2022), and especially for the distribution of phyllosphere resistomes (Li et al., 2023; Yan et al., 2021). Therefore, in addition to the environmental variables that affect ARGs abundance, the greater abundance of ARGs observed at high elevations could also be possibly resulted from intensified bacterial competition in the phyllosphere. Corroboratively, the variables of clustering coefficient in bacterial co‐occurrence network explained comparable proportion of the variances in ARG abundance when compared to the environmental variables (23.32% vs. 22.60%, respectively; Figure S13). Similarly, various previously reported core bacterial taxa in the phyllosphere, including *Sphingomonas* (Liu et al., 2023), *Methylobacterium–Methylorubrum* (Anguita‐Maeso et al., 2022) and *Erwinia* (Sahu et al., 2022), which are responsible for stress resistance and antimicrobial compound production, were enriched at high elevations (Figure S5). Among them, *Methylobacterium–Methylorubrum* participates in metabolism related to stress resistance under low nutrient conditions (Moura et al., 2021; Xie et al., 2023). *Sphingomonas* and *Erwinia* have been reported to produce antimicrobial compounds that can compete with other microbes (Burse et al., 2004; Lundberg et al., 2022). Considering that the phyllosphere is an open and complex environment, in which high elevations harbour more extreme climate conditions, such as high UV and low temperature, oxygen and water availability, it is reasonable that bacteria will compete for more nutrients and ecological niches to survive at high elevations under strong environmental stress. In addition, low temperatures, low nutrients and high UV radiation at high elevations may also promote the production of ARGs and horizontal gene transfer among phyllosphere bacterial communities (Chen, Yin, et al., 2019; Son et al., 2018; Yang et al., 2019).

The greatest number of positive associations between bacteria and ARGs was observed at high elevations (Figure 5), suggesting that phyllosphere bacteria surviving at high elevations are associated with more various types of ARGs. The phyllosphere resistomes of the Tianmu Mountain National Nature Reserve are dominated by genes associated with multidrug resistance and efflux pumps (Figure 4, S8). ARGs associated with efflux pumps, an ancient mechanism, are the primary and highly reactive defence mechanism against antibiotics or toxins (Du et al., 2018). Moreover, the overexpression of multidrug resistance and efflux pump genes has been reported in the soil microbiome in cold forests to confer resistance to environmental stress (Delgado‐Baquerizo et al., 2022). In addition to being resistant to antibiotics or toxins, ARGs associated with multidrug efflux pumps also play critical roles in intercellular trafficking among bacterial communities (Martinez, 2009; Martínez, 2008) and facilitate interactions between bacteria through bacterial communication (Aendekerk et al., 2005). The bacterial taxa detected here, including *Pseudomonas putida* (Fan et al., 2019), *Lactococcus lactis* (Alpert et al., 2003) and *Klebsiella oxytoca* (Sun et al., 2024), were reported to be capable of HGT of ARGs, possibly contributing to the tighter correlation of different taxa and ARG types observed at high elevations (Figure 4D) and thus resulting in increased resistance to antibiotic/antimicrobial threats or environmental stress for the whole community.

Nevertheless, precipitation of pollutants, including airborne particles, may also contribute to the increased abundance of ARGs in the phyllosphere at high elevations. However, according to the sampling data collected in August and September 2022 in Linan district, Hangzhou, the average monthly concentrations of the air pollutants PM 2.5 (83.5 and 83.16 μg/m^3^) and PM 10 (38.7 and 39.2 μg/m^3^; Figure S14A,B) were lower than those in other months in 2022. Moreover, the average concentrations of PM 2.5 (83.0 μg/m^3^) and PM 10 (38.9 μg/m^3^) in 2022 were lower than those in the other years (2014–2021 data from https://aqicn.org/; Figure S14C). Considering that atmospheric aerosol pollution tends to be more prevalent during the winter due to increased wet deposition, such as snowfall (Zhu et al., 2021), the precipitation of pollutants during August and September is less likely to contribute to the accumulation of phyllosphere ARGs in the national natural reserve of Tianmu Mountain. Additionally, at low and middle elevations (especially at elevations lower than 860 m), a greater fraction of the reads was assigned to the Homo genus than at high elevations, suggesting that more anthropogenic activities were occurring at low and middle elevations (Figure S14E). These results further confirmed that the higher abundance of ARGs at high elevations was unlikely to stem from human activities. Bacterial community structure and assembly were the main factors driving the distribution of phyllosphere ARGs along different elevational gradients in the Mount Tianmu National Natural Reserve.

## CONCLUSION

In conclusion, we observed an increasing abundance of ARGs in the phyllosphere along elevational gradients of mountains in national natural reserves. Greater phylogenetic overdispersion among phyllosphere bacterial communities and a greater proportion of negative associations in the bacterial co‐occurrence networks were observed at high elevations, suggesting intensified competition in phyllosphere bacteria at high elevations compared to that at low and middle elevations. The relatively greater abundance of ARGs and a greater number of ARG types that accumulated at higher elevations likely resulted from the intensified interaction among phyllosphere bacterial communities, highlighting the significant impact of bacterial community structures and assembly on ARG distribution.

## AUTHOR CONTRIBUTIONS

**Yihui Ding:** Investigation; writing – original draft; formal analysis; data curation. **Rui‐Ao Ma:** Methodology; investigation; data curation. **Ran Zhang:** Formal analysis. **Hongwei Zhang:** Formal analysis. **Jian Zhang:** Conceptualization; writing – review and editing. **Shaopeng Li:** Conceptualization; writing – review and editing. **Si‐Yu Zhang:** Conceptualization; writing – review and editing; project administration; supervision; funding acquisition.

## CONFLICT OF INTEREST STATEMENT

The authors declare that they have no conflicts of interest.

## Supporting information


**Data S1.** Supporting Information

**Data S2.** Supporting Information

## Data Availability

The raw sequencing data from 88 metagenomes were deposited in the National Centre for Biotechnology Information (NCBI) Sequence Read Archive (SRA) under BioProject PRJNA1107007.
