# Supplementary material for: Increased antibiotic resistance gene abundance linked to intensive bacterial competition in the phyllosphere across an elevational gradient
Source: Environ Microbiol Rep. 2024 Nov 21;16(6):e70042. doi: 10.1111/1758-2229.70042 (PMC11581953; doi:10.1111/1758-2229.70042)
Supplement: Supplementary file 1 — Data S1. Supporting Information [file EMI4-16-e70042-s002.docx]

**Supplementary**

**Increased antibiotic resistance gene abundance is associated with intensive bacterial competition in the phyllosphere across an elevational gradient**

Yihui Ding ^1^, Rui-Ao Ma ^1^, Ran Zhang ^1^, Hongwei Zhang ^1^, Jian Zhang ^1^, Shaopeng Li ^1^, Si-Yu Zhang ^1^*

1. School of Ecological and Environmental Sciences, East China Normal University, Shanghai, China

Corresponding author: Si-Yu Zhang

e-mail: syzhang@des.ecnu.edu.cn

FIGURE S1 Environmental conditions and leaf chemistry content.

FIGURE S2 Phylogenetic tree of plant species.

FIGURE S3 Elevation group.

FIGURE S4 Composition of phyllosphere bacterial communities along elevational gradients at the genus level.

FIGURE S5 Differential bacterial OTUs and characteristics at different elevations.

FIGURE S6 Drivers of phyllosphere bacterial community variation.

FIGURE S7 Distribution of the relative abundance of antibiotic resistance genes (ARGs) along the elevation gradient.

FIGURE S8 Characteristics of phyllosphere ARGs at different elevation gradients.

FIGURE S9 Correlations between differentially abundant bacterial OTUs in the phyllosphere and ARGs.

FIGURE S10 The relative abundance of MAGs carrying dominant ARGs at different elevations.

FIGURE S11 Composition of the ARG-bacteria interaction network.

FIGURE S12 Relative abundance of specific ARG subtypes at each elevation.

FIGURE S13 Variance partitioning analysis (VPA) results illustrating the proportion of variation in ARG abundance explained by environmental variables, the clustering coefficient of bacterial subnetworks, and unexplained variation.

FIGURE S14 Concentrations of PM2.5 and PM10 in Linan District, Hangzhou.

**MATERIALS AND METHODS**

**Estimation of soil and leaf properties**

Five equidistant sampling points were established for each plot across the elevational gradient (364–1476 m). Six soil cores (10 cm deep below the litter layer, 5 cm in diameter) were randomly extracted and mixed into a composite sample at each sampling point. The plant residues were removed, and the soil samples were air-dried and passed through 2 mm nylon. The total carbon (TC), total nitrogen (TN), total phosphate (TP), pH, and moisture contents of the soil samples were subsequently measured via the standard protocol (Liu, et al.,2021).

The collected leaf samples were processed within 12 hours of harvesting. The leaf area was estimated via ImageJ (Schneider, et al.,2012) and the R package “LeafArea” (Katabuchi,2015). In addition, harvested plant leaves were kept in paper envelopes and oven-dried at 72°C for approximately 48 hours to a constant mass. The leaf dry matter content (LDMC) was subsequently calculated as the ratio of dry leaf mass to fresh leaf mass. Before the chemical elements of the leaf samples were measured, the dried leaves were ground and passed through a 100-mesh sieve. The carbon, nitrogen, and phosphate contents of the leaves (LCC, LNC, and LPC) were estimated via the potassium dichromate method (Fu, et al.,2013), Kjeldahl method (Kacar,1994), and Mo‒Sb anti-spectrophotometer method (Lu,1999), respectively.

**Phyllosphere microbial DNA extraction and shotgun metagenomics sequencing**

Approximately 5 g of leaves per sample (15 g of leaves per sample were split into triplicate samples for elution) was transferred to conical flasks, 100 mL of 0.01 M PBS buffer (137 mM NaCl, 2.7 mM KCl, 8 mM Na_2_HPO_4_, 2 mM KH_2_PO_4_) supplemented with 0.1% tween solution was added, and the mixture was subjected to 10 min of treatment in an ultrasound bath (25°C, 100 kHz). After that, the conical flasks with the leaf samples were shaken for 1 h to facilitate the sedimentation of all the bacterial cells into the solution. The leaf washes were filtered through a 0.22 μm cellulose acetate membrane and stored at -20°C. All phyllosphere microbial DNA was extracted via the DNeasy PowerSoil Kit (Qiagen, Hilden, Germany). The DNA concentration was assessed with a NanoDrop spectrophotometer (Thermo Fisher Scientific Inc., MA, USA), and the DNA was stored at -80°C. The paired-end libraries were constructed for sequencing on the Illumina sequencing platform via the NEXTFLEX Rapid DNA-Seq platform (Bioo Scientific, Austin, TX, USA). Paired-end sequencing was performed on an Illumina NovaSeq platform (Illumina Inc., San Diego, CA, USA) at Majorbio Bio-Pharm Technology Co., Ltd. (Shanghai, China), yielding 2×150 bp reads.

**Metagenome-assembled genome (MAG) binning, taxonomic classification, and functional annotation**

Trimmed and clean paired metagenomic sequencing reads from 88 individual samples were used for metagenomic assembly with MEGAHIT version 1.2.9 with default settings (Li, et al.,2016). Contigs longer than 1500 bp were binned via MetaBAT2 version 2.15 (Kang, et al.,2019) and MaxBin2 version 2.2.7 (Wu, et al.,2016). The metagenome-assembled genomes (MAGs) were refined through bin_refinement in Metawrap version 1.2.1 (Uritskiy, et al.,2018). The resulting refined MAGs were dereplicated via dRep version 3.4.3 (Olm, et al.,2017). The completeness and contamination of MAGs were assessed via CheckM version 1.1.3 (Parks, et al.,2015), and only MAGs with completeness ≥ 50% and contamination < 10% were used for further analysis. The relative abundance of MAGs was calculated via CPG (coverage per genome equivalent). First, we used BLAT (version 2.3.4.1) to map the reads from each sample to the contigs in the MAGs with the following cut-off: identity >= 95% and alignment length >= 100 bp. Next, a script (http://enve-omics.ce.gatech.edu/enveomics/docs?t=BlastTab.seqdepth.pl) was used to calculate the coverage of each contig in the MAG, and the average coverage of all contigs in the MAG was used as the coverage of the MAG. The genome equivalents, which were equal to the total bp sequenced/average genome size in bp, for each sample were calculated via MicrobeCensus v1.1.0 (Nayfach, et al.,2015). Finally, the abundance of the MAG was normalized to that of the CPG. The open reading frames (ORFs) of the MAGs were predicted via Prodigal (version 2.6.3) (Hyatt, et al.,2010) with the parameter ‘-p meta’ for functional gene annotation. Taxonomic annotations for MAGs were performed via the module classify_wf in GTDB-Tk (https://github.com/Ecogenomics/GTDBTk) (version 2.1.0), and the module inferred from GTDB-Tk was used for phylogenetic tree construction. The phylogenetic tree of the MAGs was visualized with iTOL (<https://itol.embl.de/>).

**Estimation of the effects of environmental factors on bacterial communities and ARG abundances**

Canonical correspondence analysis (CCA) and variance partitioning analysis (VPA) were used to evaluate the contributions of multiple factors, including host factors (i.e., leaf functional traits and phylogeny) and abiotic factors, including soil properties and geographic factors, to the variation in bacterial community structure. Differences in the structure of bacteria and ARGs among samples were analysed through the PERMANOVA test, which employs the adonis2 function with 999 permutations, also in the “vegan” package. The contribution of bacterial communities to changes in ARGs along elevational gradients was determined through the Procrustes test with the “vegan” and “labdsv” packages (Roberts, et al.,2016). Mantel tests were also performed via the “vegan” package for estimation of the effects of multiple factors on the variation in bacterial communities and ARG abundance. For the Mantel test, the first axis of the PCA of soil properties and leaf traits and the first axis of the NMDS of bacterial composition were used to explore the contribution to the variation in the phyllosphere resistomes along the elevational gradient. All the statistical analyses were performed in the R environment (version 4.2.3), and the results were visualized with the “ggplot2” package (Wickham,2011). All *P* values for multiple comparisons were corrected via the Benjamini–Hochberg procedure (Benjamini, et al.,1995).

**Results**

**Description of climate, soil, and leaf properties and division of low, medium, and high elevational groups**

The average monthly air temperature decreased along the elevational gradient, ranging from 20.47°C to 14.93°C. The highest air temperature was observed at the lowest elevation (364 m), whereas the lowest air temperature was observed at the highest elevation (1476 m; Figure S1A, Table S1). In soils, there was a hump-shaped elevational pattern for the contents of moisture, carbon, and nitrogen (Figure S1D, E, Table S1), whereas the phosphorus content (Figure S1 F, Table S1) was greatest at 787 m. In addition, the mountain ecosystem exhibited a U-shaped pattern for soil pH (Figure S1C, Table S1). Twenty-three dominant plant species, which were affiliated with 11 plant families, were collected at 10 elevations. Among them, *Lauraceae* was the most distant plant family related to the other plant families (Figure S2). According to the NMDS plot based on the Bray‒Curtis distance of soil properties, air temperature, and leaf traits, a clear separation pattern was revealed for the samples collected from low elevations (364–664 m; 34 samples) from the middle (787-950 m; 27 samples) and high elevations (1113 – 1476 m; 27 samples; Figure S3A). Similarly, according to the NMDS plot, significant variances in the soil properties, air temperature, and leaf traits were also revealed at middle and high elevations (Figure S3B). In addition, for the middle and high elevations, the vegetation type was also taken into consideration, i.e., the middle elevations are dominated by evergreen and deciduous broad-leaved mixed forest, whereas the high elevations mostly consist of deciduous broad-leaved forest, as previously reported (Da, et al.,2009).

**Co-occurrence patterns of bacterial communities at different elevational gradients**

We constructed a bacterial interaction network for each elevation gradient, and the bacterial OTUs in the networks were affiliated mainly with 5 phyla (*Pseudomonadota*, *Actinomyceota*, *Bacteroidota*, *Acidobacteriota*, and *Bacillota*) (Figure 3A). In the co-occurrence network at high elevations, the OTUs with negative associations were associated mainly with the genera *Enterobacter*, *Methylobacterium-Methylorubrum*, *Escherichia-Shigella* and *Methylobacterium-Methylorubrum* (Figure 3A, Table S14). Additionally, the modularity of the three elevation gradients was greater than 0.4, suggesting great modularity at each elevation (Figure 3A).

**SEMs illustrating factors contributing to variance in bacterial communities**

The structural equation model explored the direct and indirect factors shaping the phyllosphere bacterial communities (Figure 6C). The results revealed that environmental factors, plant attributes (leaf traits and plant phylogeny), and soil properties explained only a limited portion of the variation in phyllosphere bacterial communities along the elevational gradient (*R*^2^ = 0.05). Air temperature, plant phylogeny, and plant traits had direct effects on the variations in the phyllosphere bacterial communities. In addition, air temperature was the main factor influencing the structure of phyllosphere bacterial communities through multiple pathways, including both directly influencing and influencing bacterial communities through plants.

**Abundance and diversity of phyllosphere ARGs at different elevational gradients**

Multidrug resistance was the dominant ARG type in the natural mountain ecosystem, accounting for 32.4%-83.2% of the phyllosphere samples. The relative abundances of multidrug resistance genes were 65.7%, 69.5%, and 67.3% at low, middle, and high elevations, respectively (Figure S8A). The following ARG types were detected: MLS, bacitracin beta-lactam, etc. (Figure S8A). Most of the ARG subtypes, such as the multidrug transporters *emrB*, *mdtK*, and *TolC*, are multidrug resistant (Figure S8A). There was no significant difference in the richness of antibiotic resistance genes (Wilcoxon rank-sum test, *P* > 0.05; Kruskal‒Wallis test, *P* > 0.05) (Figure S8B). The relative abundances of bacitracin, kasugamycin, polymyxin, and fosmidomycin resistance genes at high elevations were not significantly greater than those at low and middle elevations (Wilcox rank-sum test, *P* > 0.05). In addition, compared with those at other elevations, the relative abundances of genes encoding antibiotic target alterations, mutations, efflux pump RND family members, and regulator resistance mechanisms were not significantly greater at high elevations (Wilcoxon rank-sum test, *P* > 0.05).

**Correlation between the abundance of ARGs and significantly differentially abundant bacteria**

Eleven phyllosphere bacterial OTUs (i.e., OTU20112: g_*Raoultella*, OTU12223: f_*Yersiniaceae*, and OTU17940: o_*Enterobacterales*) with significantly enriched abundance at high elevations presented a strong positive correlation with ARG abundance (Figure S9A). Moreover, most of the differentially abundant bacteria (i.e., OTU18946 g_*Pseudomonas*, OTU915 g_*Massilia*, OTU16296 g_*Massilia*, and OTU529 s_*Methylobacterium*_sp.) ), which was negatively correlated with ARG abundance, decreased at high elevations (Figure S5D, S9A). OTU14933 and OTU8861 were enriched at high elevations but had negative correlations with ARG abundance (Figure S5D, S9A). In addition, eight differentially abundant bacterial OTUs were significantly correlated with ARG richness. Among these bacterial OTUs, OTU8524 (g_*Ralstonia*) was significantly negatively correlated with ARG richness, and the phyllosphere bacterial OTUs (OTU8636 g_*Stenotrophomonas*, OTU19107 g_*Pseudomonas*, OTU18946 g_*Pseudomonas*, OTU14331 o_*Enterobacterales*, and OTU18121 g_*Amnibacterium*) were positively correlated with ARG richness.

Additionally, many differentially abundant bacterial OTUs were significantly correlated with ARG type (Figure S9B). The bacterial taxa belonging to *Pseudomonadota* (i.e., *Serratia*, *Raoultella*, *Klebsiella*, *Cedecea*, *Pantoea*, and *Erwinia*) had strong positive correlations with ARGs, including fosmidomycin, fosfomycin, MLS, vancomycin, and multidrug resistance genes. Additionally, the abundances of the genera *Massilia* and *Methylobacterium-Methylorubrum* were significantly negatively correlated with the abundance of ARGs. Moreover, vancomycin and trimethoprim ARG types were positively related to bacterial OTUs affiliated with *Actinomycetota* (such as *Pseudarthrobacter*), and most of the other ARG types had significantly negative relationships with differentially abundant bacterial OTUs.

**References**

Liu L, Zhu K, Krause S M B, Li S, Wang X, Zhang Z, et al. Changes in assembly processes of soil microbial communities during secondary succession in two subtropical forests. *Soil Biol. Biochem.* 2021; 154.

Schneider C A, Rasband W S and Eliceiri K W. NIH Image to ImageJ: 25 years of image analysis. *Nat. Methods*. 2012; 9:671-75.

Katabuchi M. LeafArea: an R package for rapid digital image analysis of leaf area. *Ecol. Res.* 2015; 30:1073-77.

Fu Y and Sun Y. A study of the determination of organic carbon of vegetation. *World Forestry Research*. 2013; 26:24-30.

Kacar B. Bitki ve toprağın kimyasal analizleri. Ankara Ünİversitesi Ziraat Fakültesi Eğitim, Araştırma ve Geliştirme Vakfı. 1994

Lu R. Soil and agro-chemical analytical methods. *China Agricultural Science and Technology Press, Beijing*. 1999; 107:146-95.

Li D, Luo R, Liu C M, Leung C M, Ting H F, Sadakane K, et al. MEGAHIT v1.0: A fast and scalable metagenome assembler driven by advanced methodologies and community practices. *Methods*. 2016; 102:3-11.

Kang D D, Li F, Kirton E, Thomas A, Egan R, An H, et al. MetaBAT 2: an adaptive binning algorithm for robust and efficient genome reconstruction from metagenome assemblies. *PeerJ*. 2019; 7:e7359.

Wu Y-W, Simmons B A and Singer S W. MaxBin 2.0: an automated binning algorithm to recover genomes from multiple metagenomic datasets. *Bioinformatics*. 2016; 32:605-07.

Uritskiy G V, DiRuggiero J and Taylor J. MetaWRAP—a flexible pipeline for genome-resolved metagenomic data analysis. *Microbiome*. 2018; 6:1-13.

Olm M R, Brown C T, Brooks B and Banfield J F. dRep: a tool for fast and accurate genomic comparisons that enables improved genome recovery from metagenomes through dereplication. *ISME J.* 2017; 11:2864-68.

Parks D H, Imelfort M, Skennerton C T, Hugenholtz P and Tyson G W. CheckM: assessing the quality of microbial genomes recovered from isolates, single cells, and metagenomes. *Genome Res.* 2015; 25:1043-55.

Nayfach S and Pollard K S. Average genome size estimation improves comparative metagenomics and sheds light on the functional ecology of the human microbiome. *Genome Biol*. 2015; 16:51.

Hyatt D, Chen G-L, LoCascio P F, Land M L, Larimer F W and Hauser L J. Prodigal: prokaryotic gene recognition and translation initiation site identification. *BMC Bioinf.* 2010; 11:1-11.

Kembel S W, Cowan P D, Helmus M R, Cornwell W K, Morlon H, Ackerly D D, et al. Picante: R tools for integrating phylogenies and ecology. *Bioinformatics*. 2010; 26:1463-64.

Webb C O, Ackerly D D, McPeek M A and Donoghue M J. Phylogenies and community ecology. *Annu. Rev. Ecol. Evol. Syst.* 2002; 33:475-505.

Li D. rtrees: an R package to assemble phylogenetic trees from megatrees. *Ecography*. 2023; e06643.

Paradis E, Claude J and Strimmer K. APE: analyses of phylogenetics and evolution in R language. *Bioinformatics*. 2004; 20:289-90.

Roberts D W and Roberts M D W. Package ‘labdsv’. *Ordination and Multivariate*. 2016; 775:1-68.

Wickham H. ggplot2. *Wiley Interdiscip. Rev. Comput. Stat.* 2011; 3:180-85.

Benjamini Y and Hochberg Y. Controlling the false discovery rate: a practical and powerful approach to multiple testing. *J. R. Stat. Soc. Series B Stat. Methodol.* 1995; 57:289-300.

Da L-J, Kang M-M, Song K, Shang K-K, Yang Y-C, Xia A-M, et al. Altitudinal zonation of human-disturbed vegetation on Mt. Tianmu, eastern China. *Ecol. Res.* 2009; 24:1287-99.


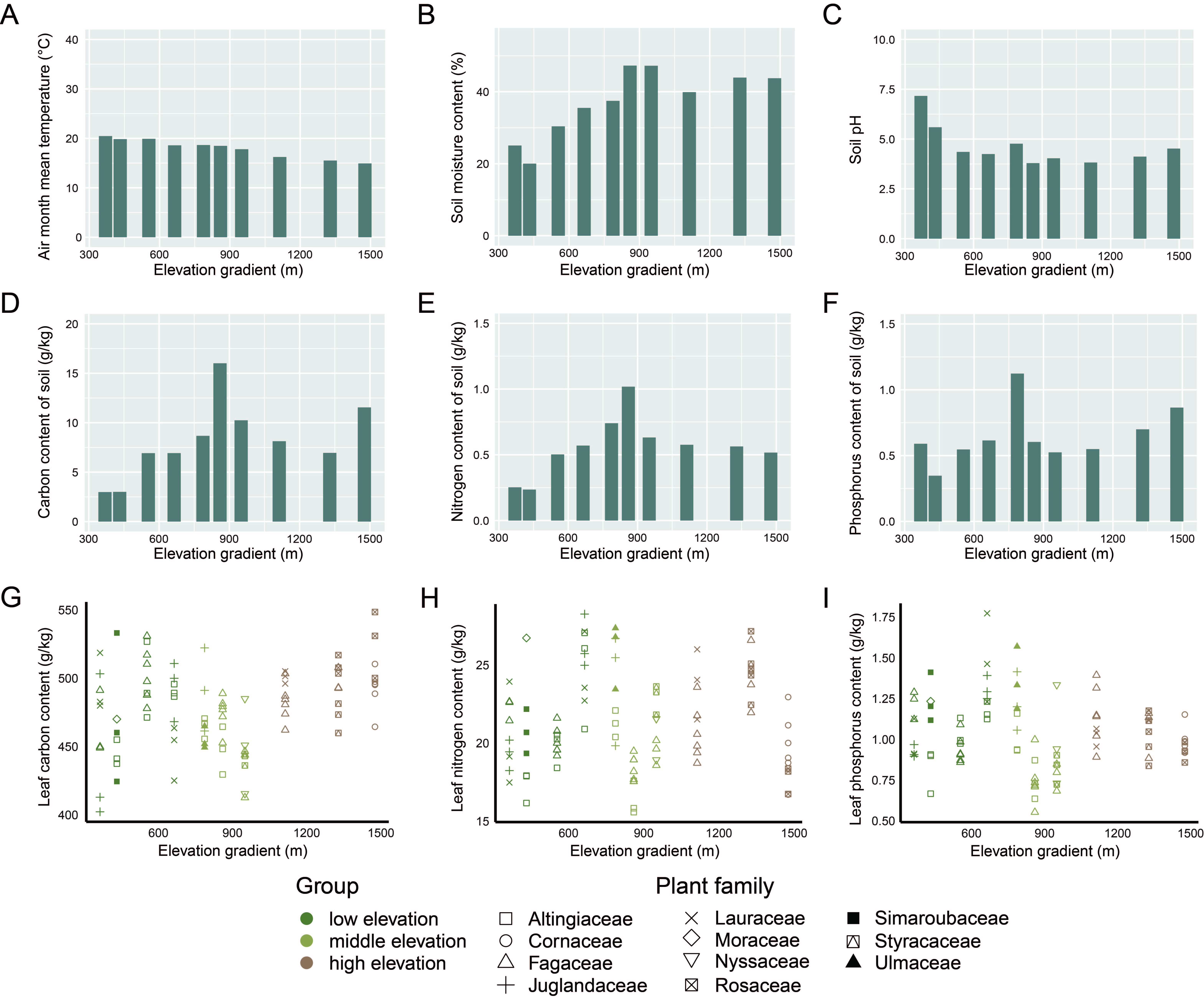


**FIGURE S1 Environmental conditions and leaf chemistry content.** (A) Monthly air temperature along the elevation gradient in September 2020. (B) Soil moisture content; (C) soil pH; (D) carbon, (E) nitrogen, and (F) phosphorus contents of the soil along the elevational gradient. Leaf carbon (G), nitrogen (H), and phosphorus (I) contents along the elevational gradient. The color represents the elevational gradient, and the shape represents the plant family.


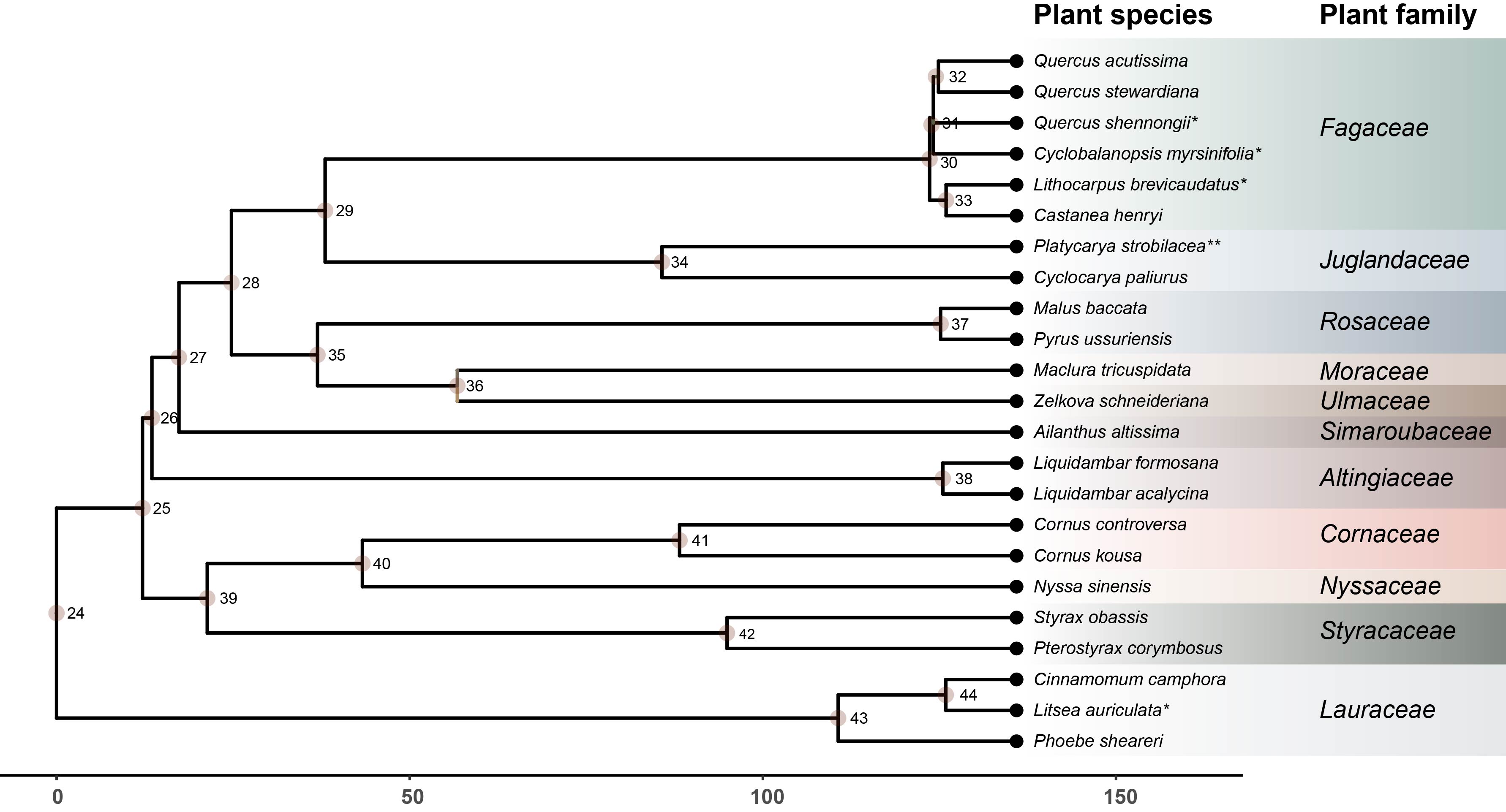


**FIGURE 2** **Phylogenetic tree of plant species.** The color represents the different plant families.


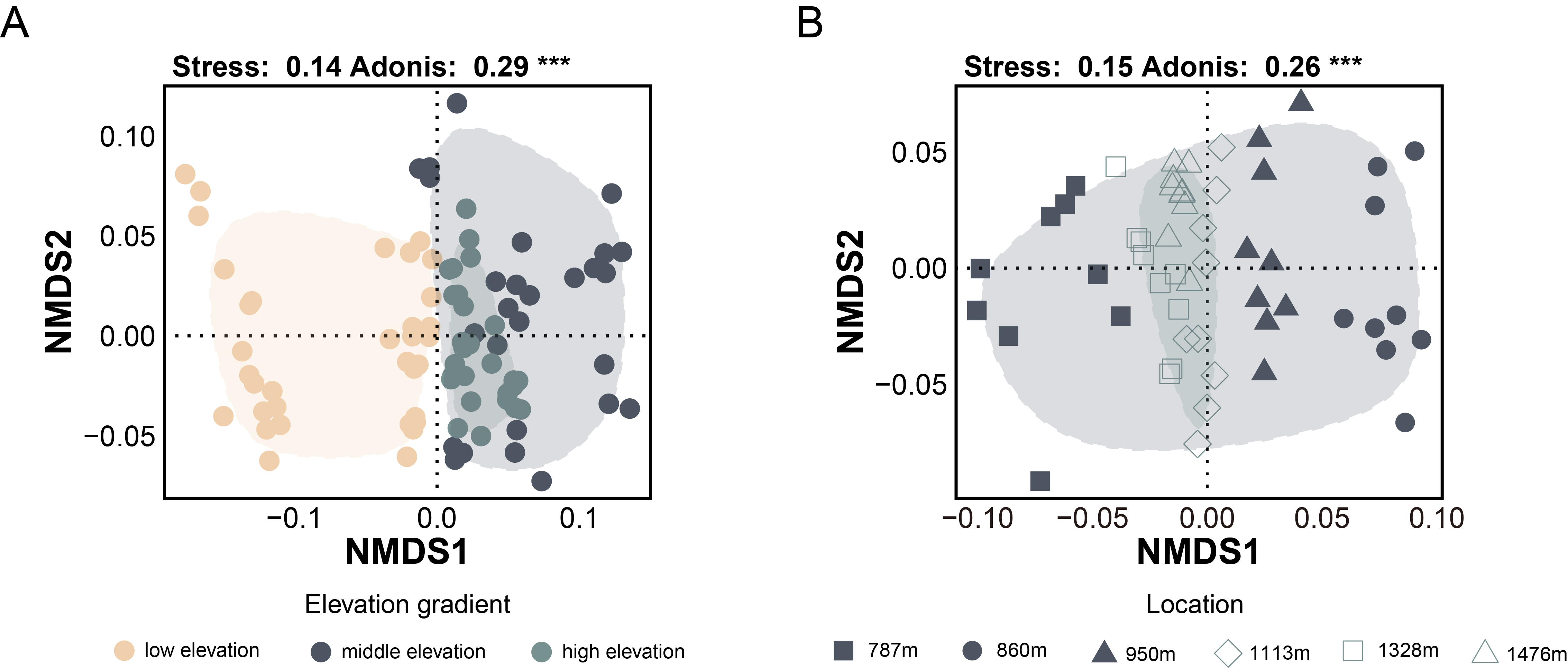


**FIGURE S3. Elevation group.** Nonmetric multidimensional scaling (NMDS) is based on Bray‒Curtis dissimilarity. The symbol “***” indicates PERMANOVA: *P* < 0.001.


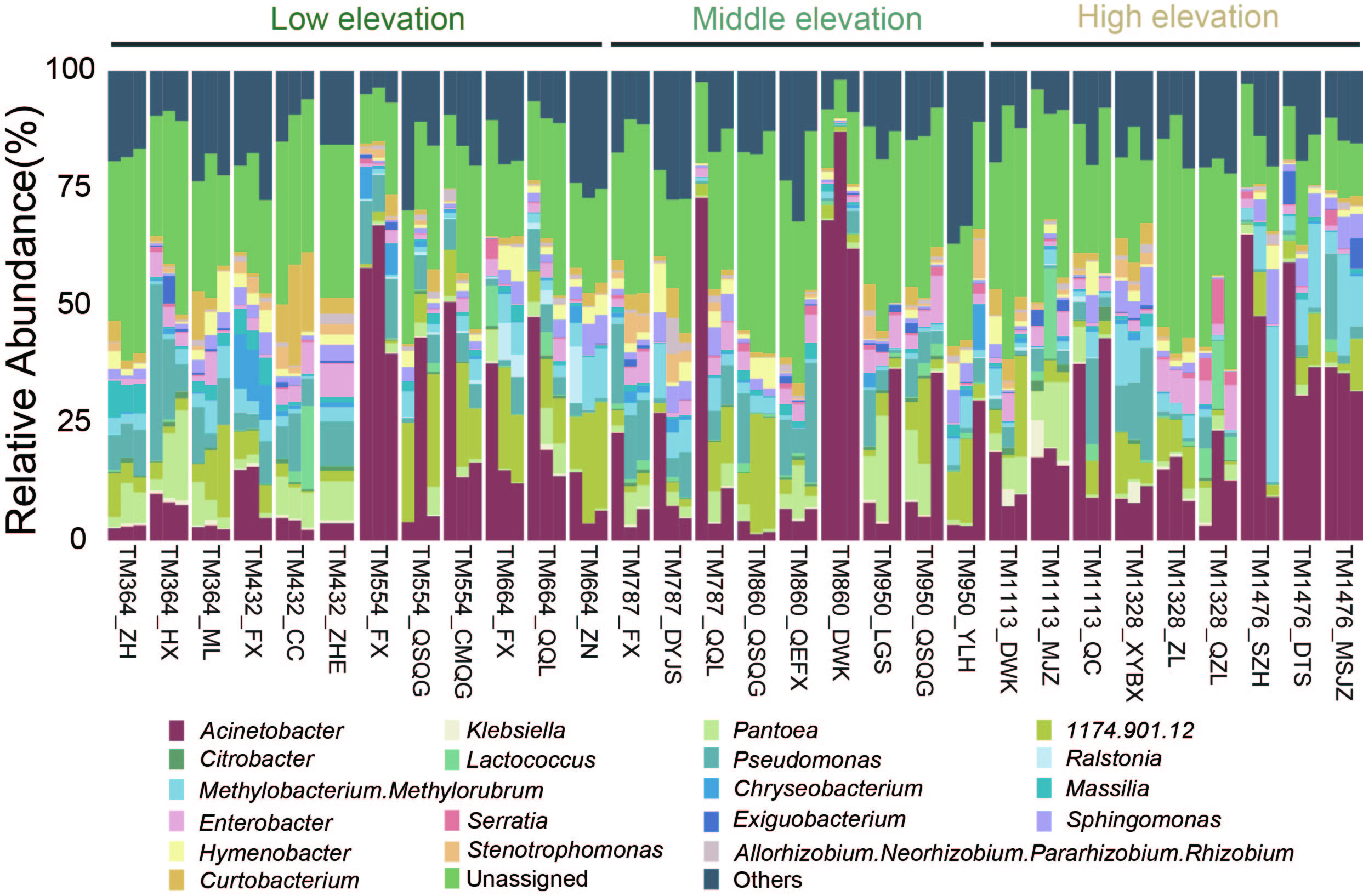


**FIGURE S4** **Composition of phyllosphere bacterial communities along elevational gradients at the genus level**.


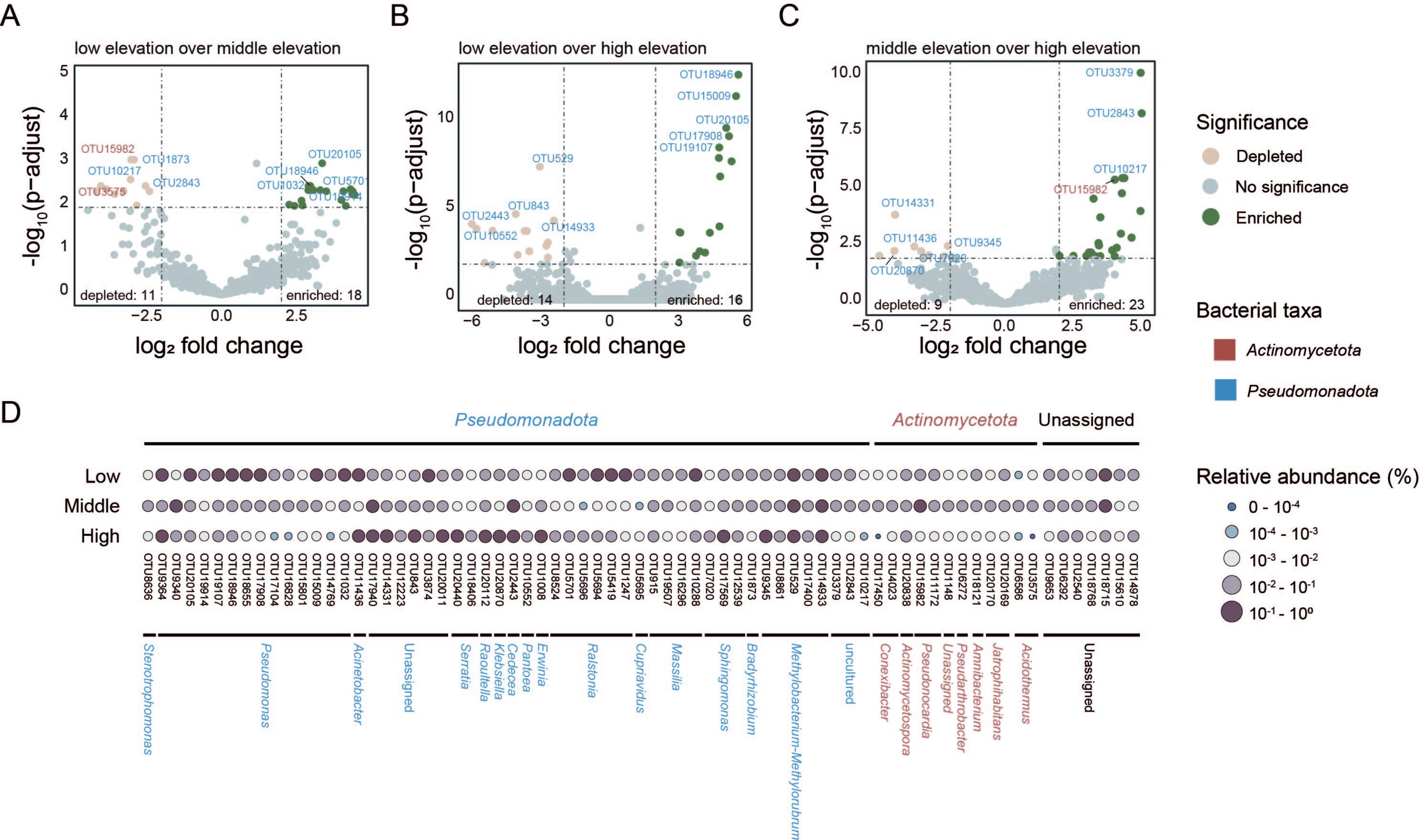


**FIGURE S5** **Differential bacterial OTUs and characteristics at different elevations.** Differential bacterial OTUs between paired elevation gradients and the relative abundance of differential bacterial OTUs between paired elevations (A) low elevation over middle elevation, (B) low elevation over high elevation, and (C) middle elevation over high elevation. (D) The relative abundance of differentially abundant bacterial OTUs according to DESeq2 analysis.


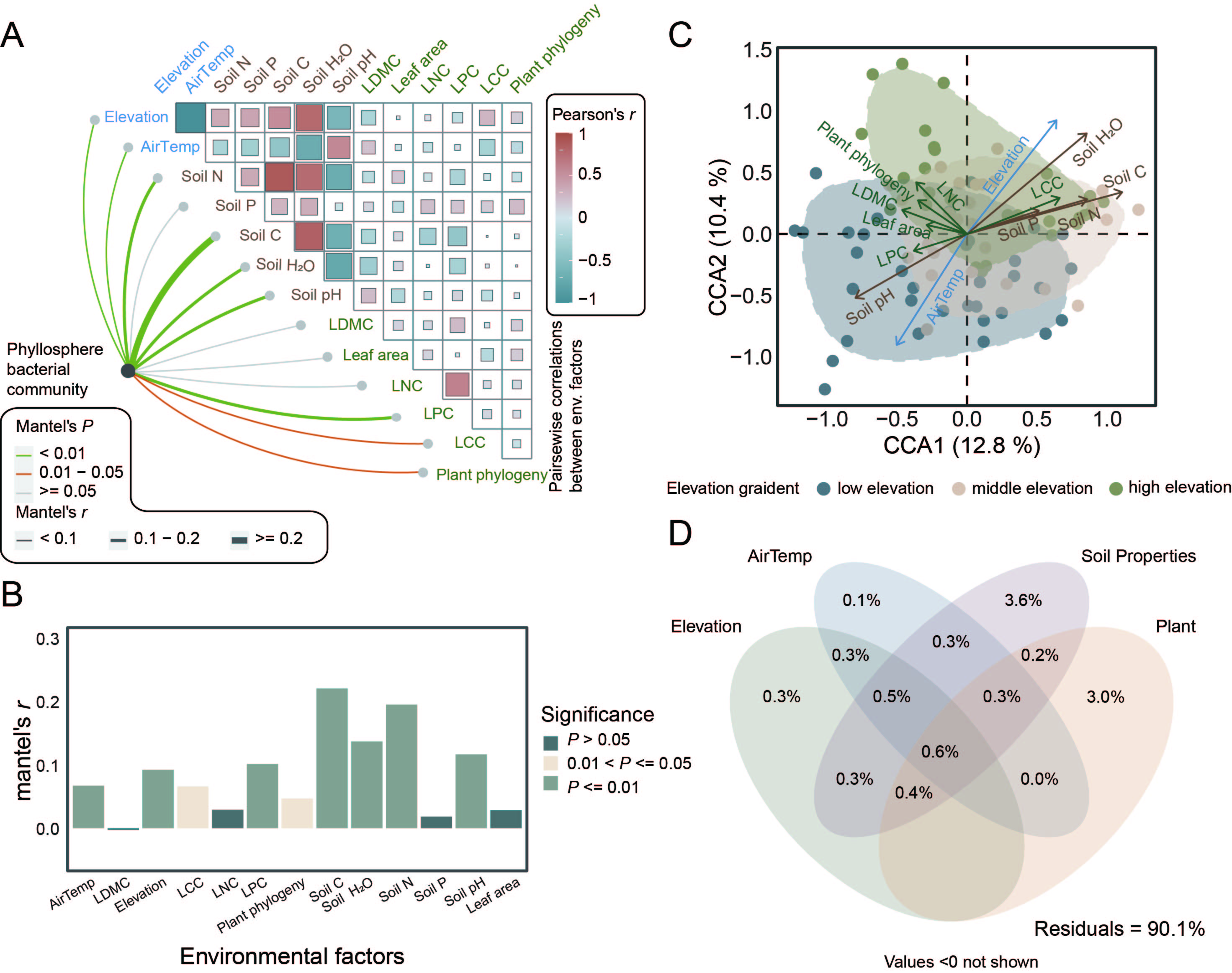


**FIGURE S6** **Drivers of phyllosphere bacterial community variation.** Environmental conditions contribute to the variation in phyllosphere bacterial communities along elevational gradients according to (A) Mantel’s test, (B) Mantel’s *r* value for different factors distributed to phyllosphere bacterial communities, (C) canonical correspondence analysis (CCA), and (D) variation partition analysis (VPA).


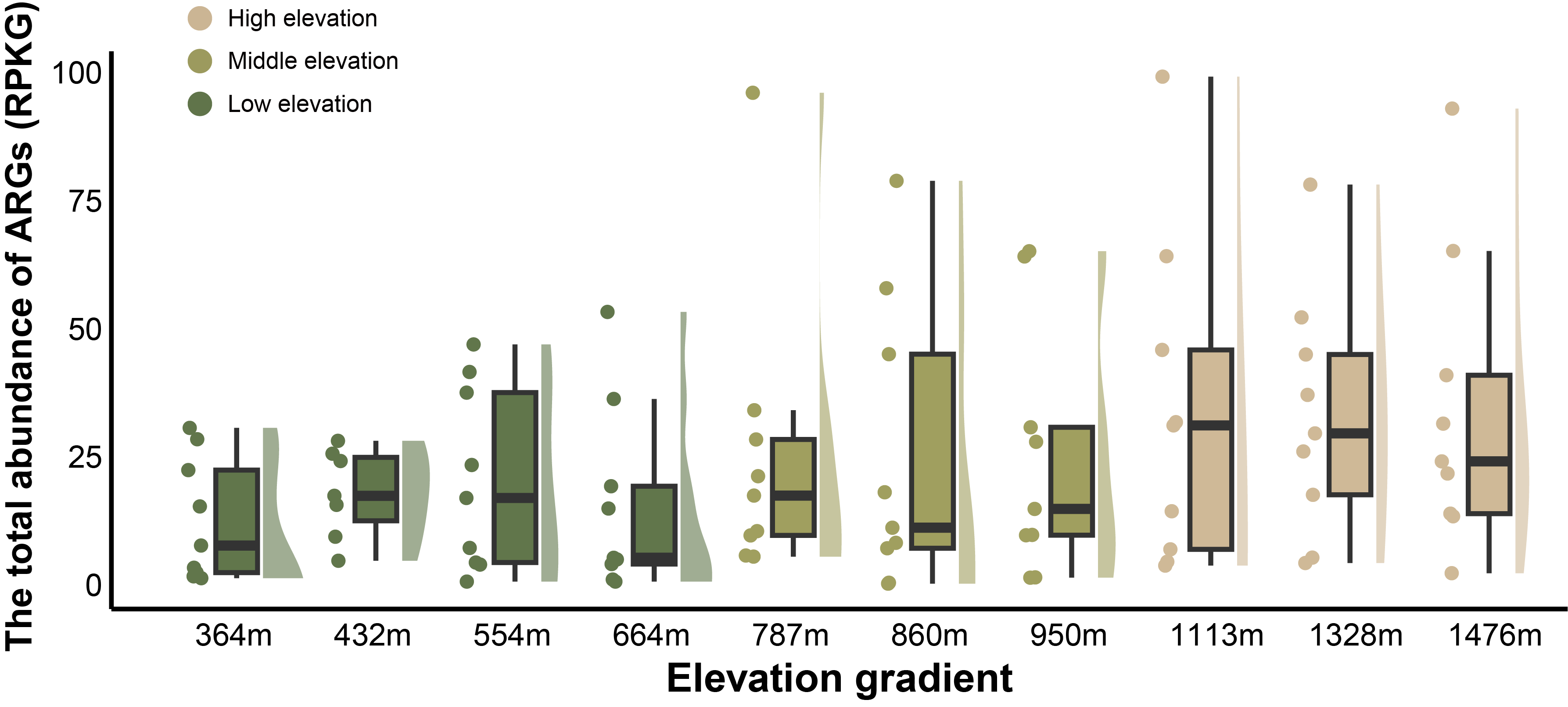


**FIGURE S7 Distribution of the relative abundance of antibiotic resistance genes (ARGs) along the elevation gradient.** The different colours represent the elevational groups.


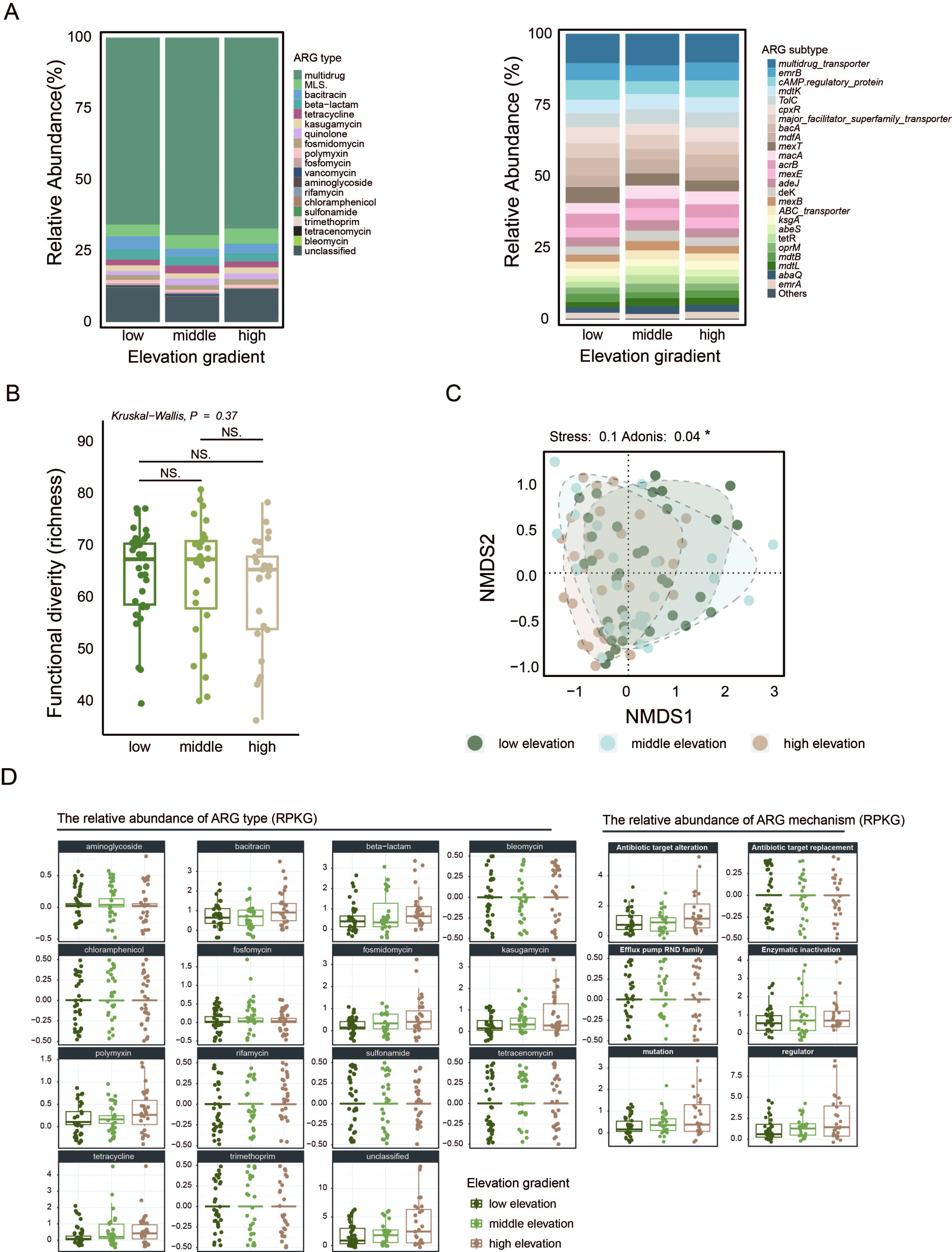


**FIGURE S8 Characteristics of phyllosphere ARGs at different elevation gradients.** (A) Composition of ARG types and subtypes. Alpha diversity (B) was based on the richness index, and beta diversity (C) was based on Bray‒Curtis dissimilarity. NS. stands for “No Significance”. The symbol “*” indicates PERMANOVA: *P* < 0.001. (D) The relative abundance of ARG types and mechanisms.


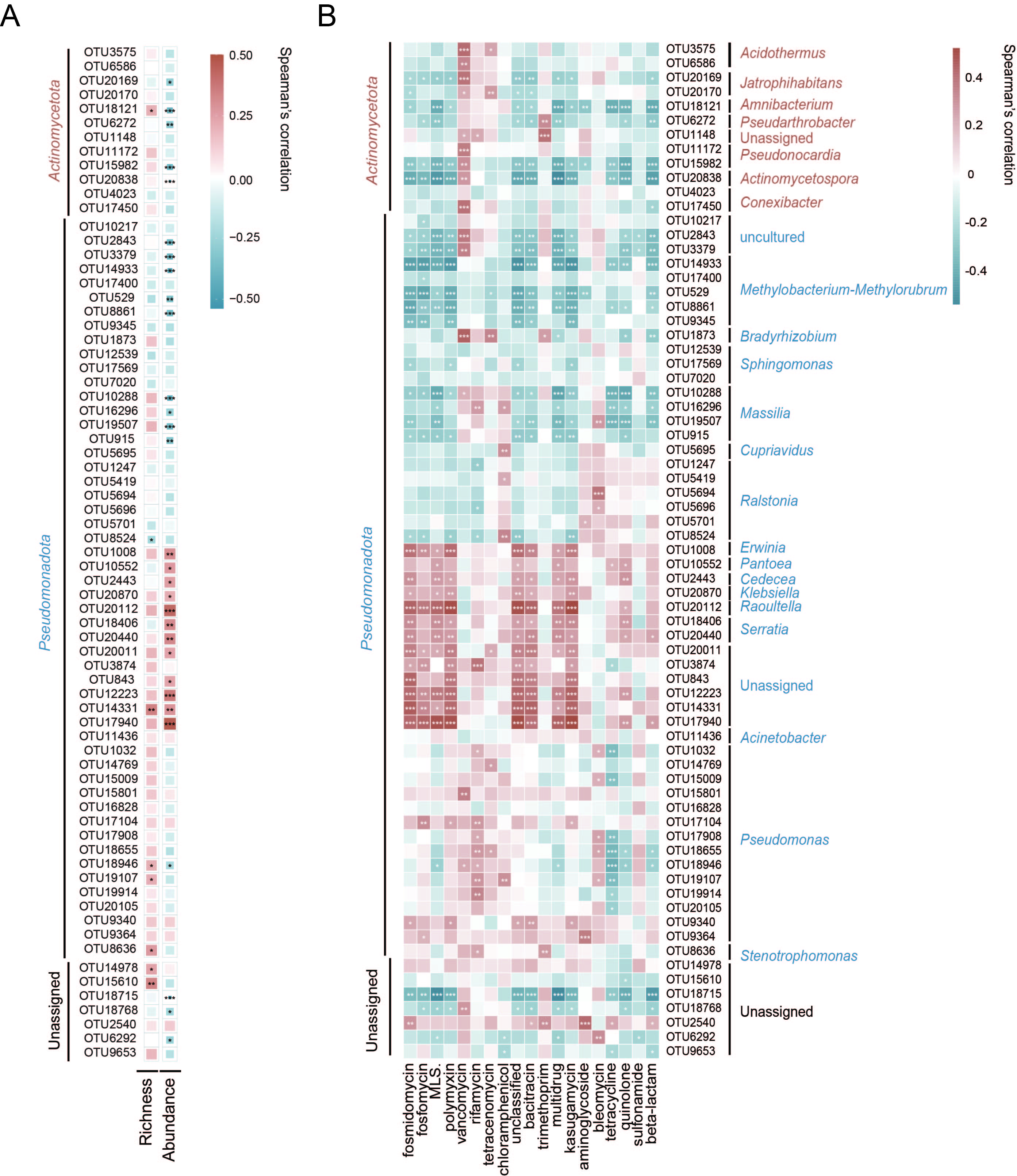


**FIGURE S9 Correlations between differentially abundant bacterial OTUs in the phyllosphere and ARGs.** (A) Spearman’s rank correlation of differentially abundant bacterial OTUs with ARG abundance and richness. (B) Spearman’s rank correlation of differentially abundant bacterial OTUs with ARG types. “*”, “**”, and “**” indicate *P* < 0.05, *P* < 0.01, and *P* < 0.001, respectively.


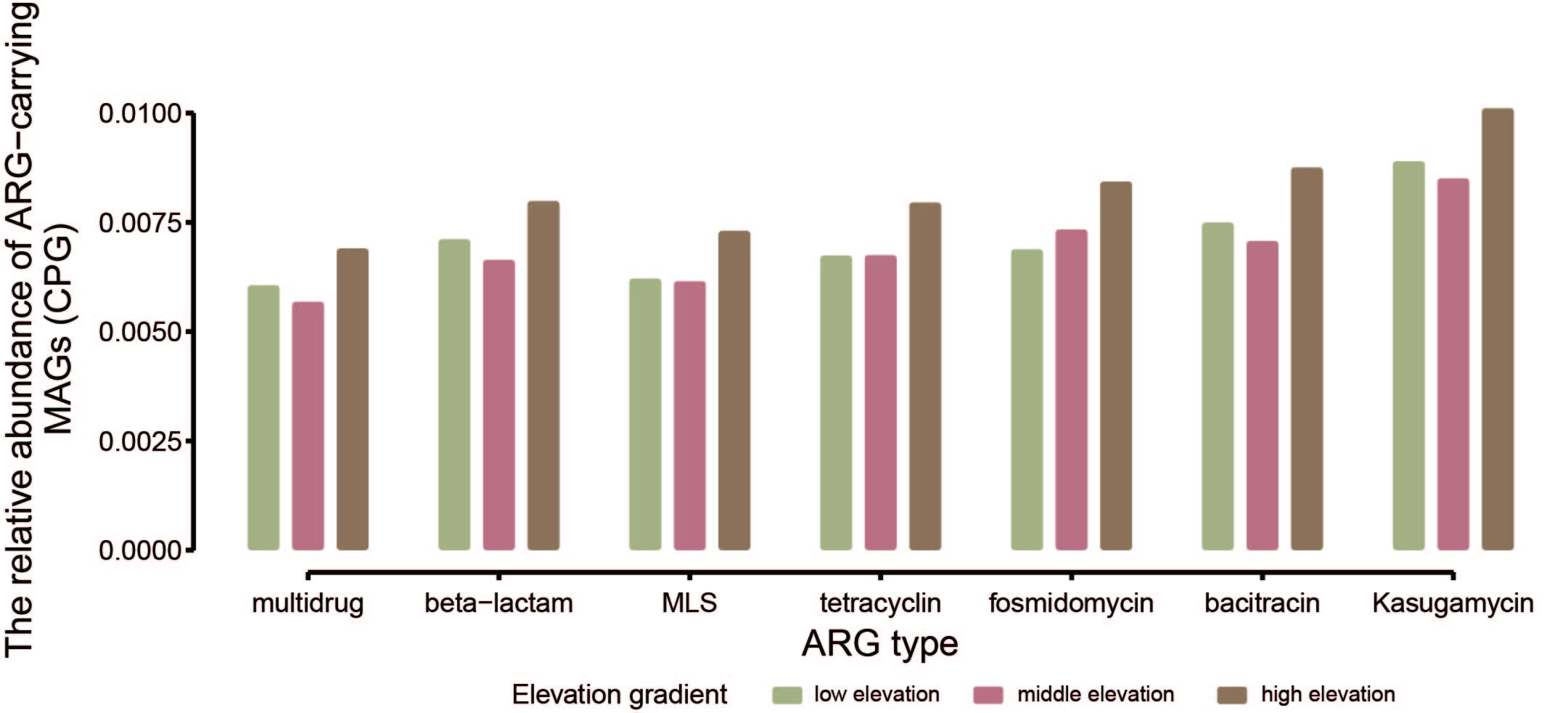


**FIGURE S10 The relative abundance of MAGs carrying dominant ARGs at different elevations.** The different colours represent different elevations.


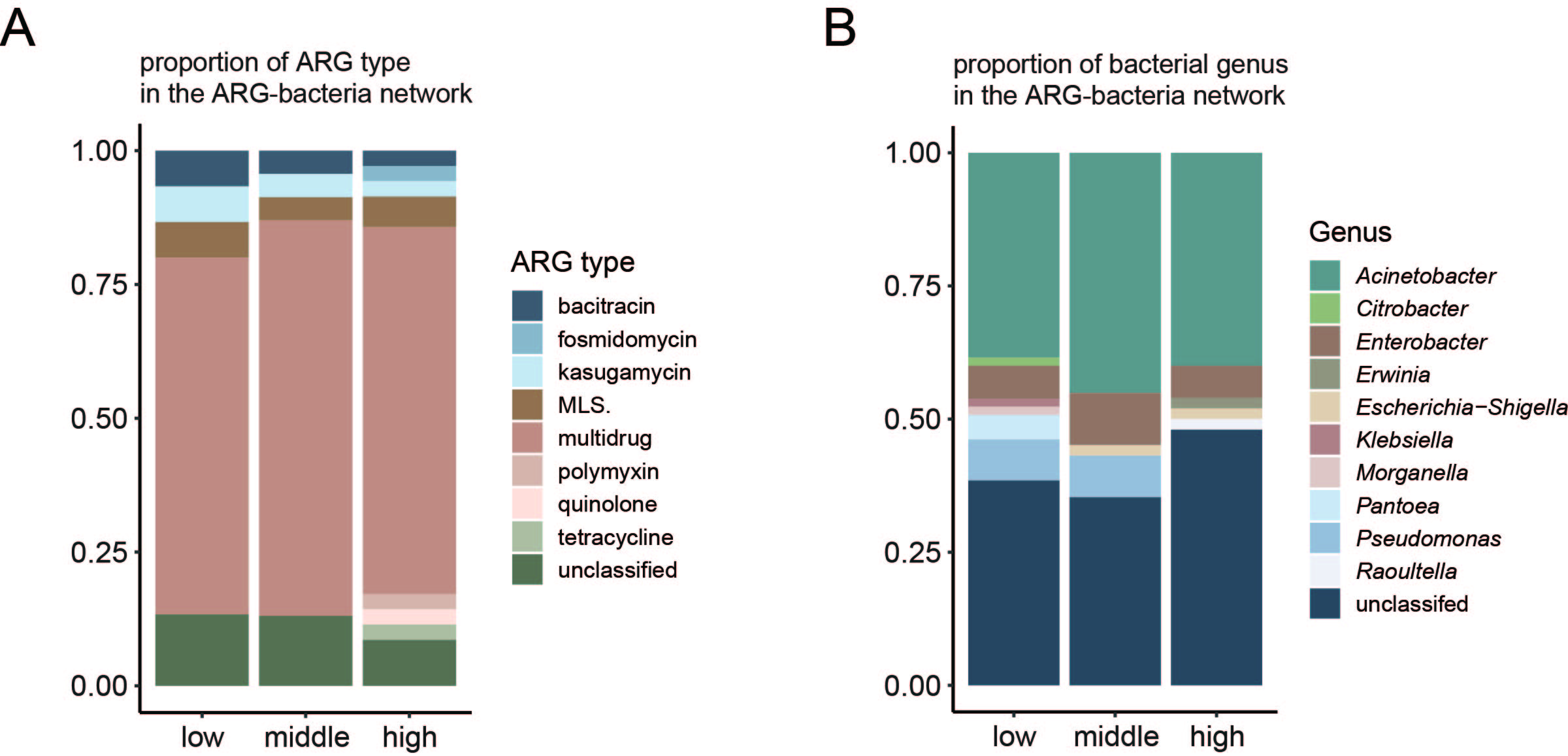


**FIGURE S11 Composition of the ARG-bacteria** interaction network. (A) Proportion of ARG types in the ARG-bacteria networks. (B) The proportions of bacterial genera in the interkingdom networks.


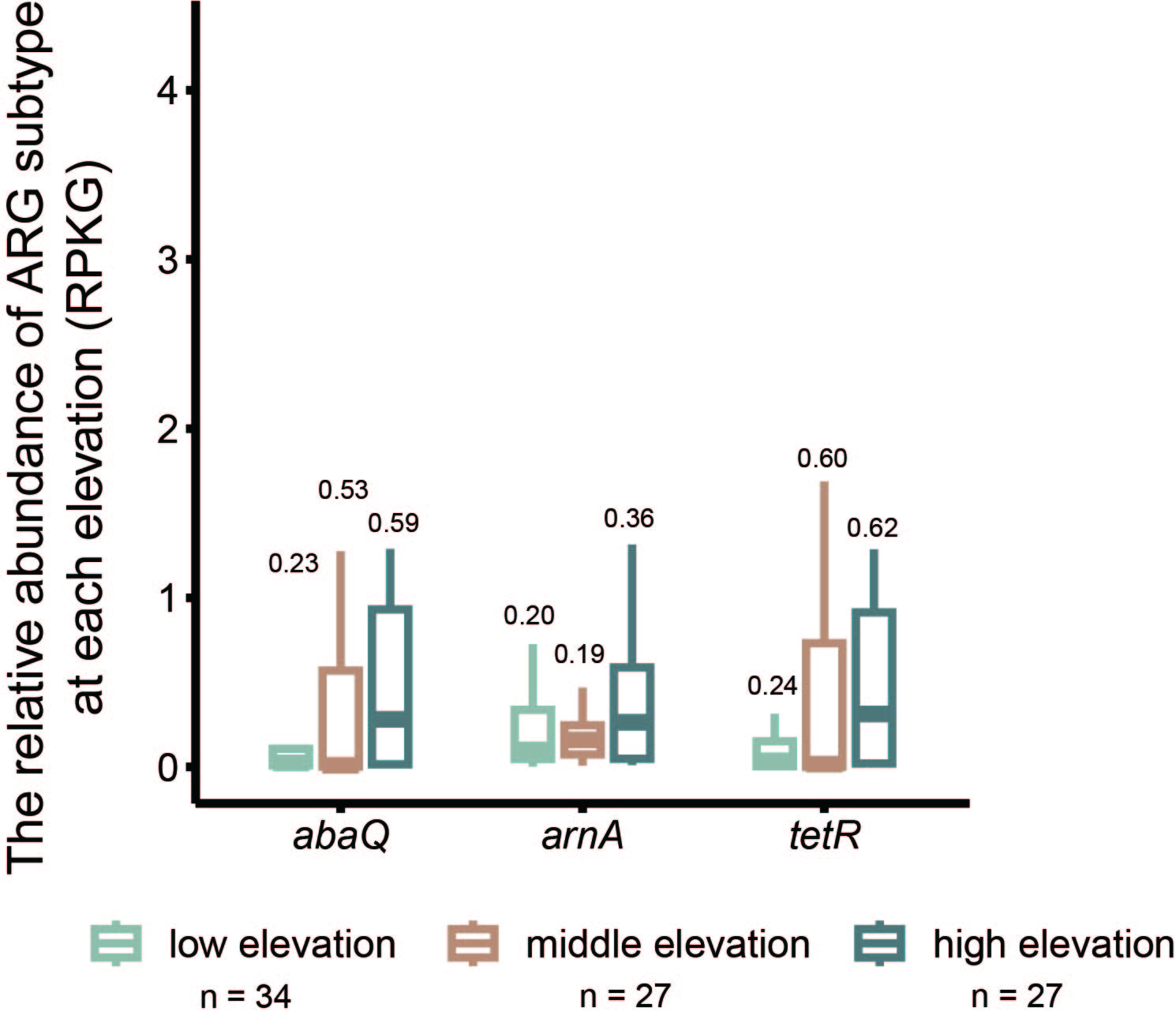


**FIGURE** **S12 Relative abundance of specific ARG subtypes at each elevation.** The numbers above the boxes represent the mean relative abundances of the ARG subtypes at each elevation. The numbers below the legend are the sample volumes per elevation.


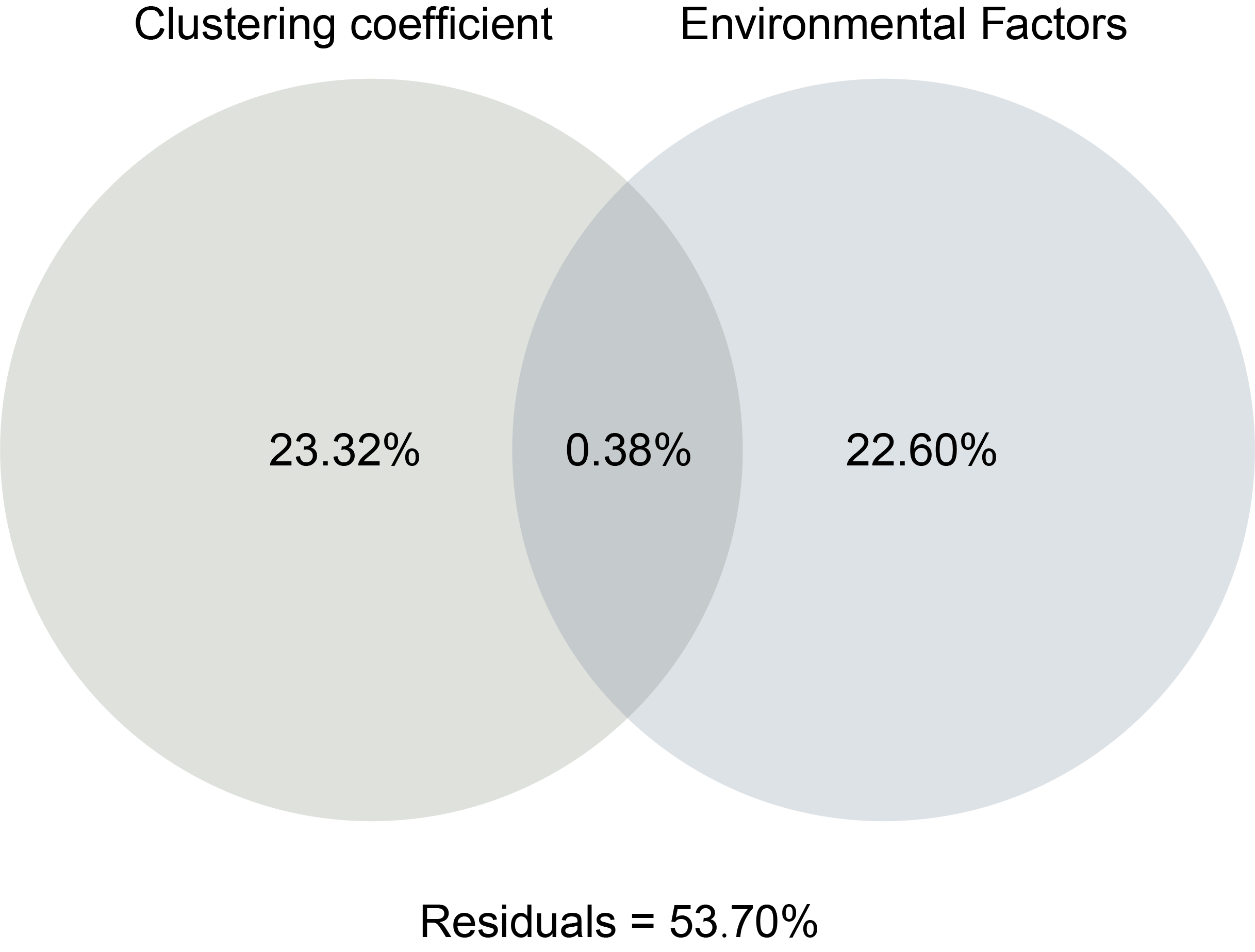


**FIGURE S1****3 Variance partitioning analysis (VPA) results illustrating the proportion of variation in ARG abundance explained by environmental variables, the clustering coefficient of bacterial subnetworks, and unexplained variation.** The environmental variables included leaf traits (leaf surface area, leaf dry matter content, leaf carbon content, leaf nitrogen content, and carbon phosphate content), plant phylogeny (PCNM1), soil properties (soil moisture content, soil pH, total carbon, total nitrogen, and total phosphate content), and air temperature.


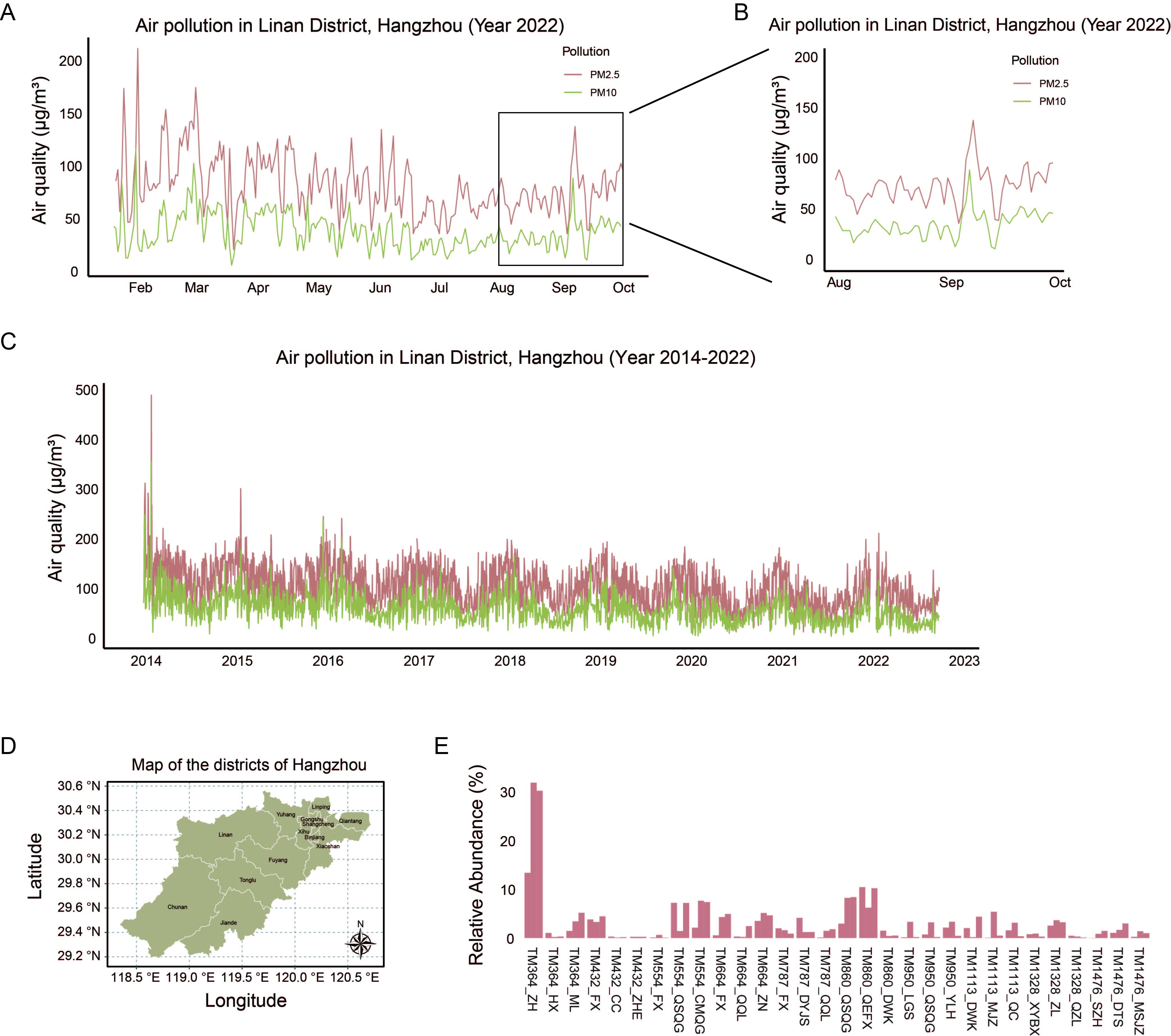


**FIGURE S14 Concentrations of PM2.5 and PM10 in Linan District, Hangzhou.** (A) Concentrations of PM2.5 and PM10 in 2022. (B) Concentrations of PM2.5 and PM10 in August and September 2022. (C) PM2.5 and PM10 concentrations from 2014--2022. (D) Map of the districts of Hangzhou. (E) The fraction of reads assigned to *Homo sapiens* (based on the genus level). The red line indicates PM 2.5, and the green line indicates PM 10.
